# Colon cancer-specific diagnostic and prognostic biomarkers based on genome-wide abnormal DNA methylation

**DOI:** 10.18632/aging.103874

**Published:** 2020-11-17

**Authors:** Yilin Wang, Ming Zhang, Xiaoyun Hu, Wenyan Qin, Huizhe Wu, Minjie Wei

**Affiliations:** 1 Department of Pharmacology, School of Pharmacy, China Medical University, Shenyang110122, Liaoning Province, P. R. China; 2 Liaoning Key Laboratory of Molecular Targeted Anti-Tumor Drug Development and Evaluation, Liaoning Cancer Immune Peptide Drug Engineering Technology Research Center, Key Laboratory of Precision Diagnosis and Treatment of Gastrointestinal Tumors, Ministry of Education, China Medical University, Shenyang110122, Liaoning Province, P. R. China

**Keywords:** COAD, DMP, pan-cancer, diagnosis, prognosis

## Abstract

Abnormal DNA methylation is a major early contributor to colon cancer (COAD) development. We conducted a cohort-based systematic investigation of genome-wide DNA methylation using 299 COAD and 38 normal tissue samples from TCGA. Through conditional screening and machine learning with a training cohort, we identified one hypomethylated and nine hypermethylated differentially methylated CpG sites as potential diagnostic biomarkers, and used them to construct a COAD-specific diagnostic model. Unlike previous models, our model precisely distinguished COAD from nine other cancer types (e.g., breast cancer and liver cancer; error rate ≤ 0.05) and from normal tissues in the training cohort (AUC = 1). The diagnostic model was verified using a validation cohort from The Cancer Genome Atlas (AUC = 1) and five independent cohorts from the Gene Expression Omnibus (AUC ≥ 0.951). Using Cox regression analyses, we established a prognostic model based on six CpG sites in the training cohort, and verified the model in the validation cohort. The prognostic model sensitively predicted patients’ survival (*p* ≤ 0.00011, AUC ≥ 0.792) independently of important clinicopathological characteristics of COAD (e.g., gender and age). Thus, our DNA methylation analysis provided precise biomarkers and models for the early diagnosis and prognostic evaluation of COAD.

## INTRODUCTION

Colon cancer (COAD) is one of the most common malignancies and the third most common cause of tumor-related mortality worldwide [1]. The survival of COAD patients highly depends on the stage of the tumor; thus, the diagnosis of COAD at an early stage can greatly enhance patients’ chances of survival [2]. The five-year survival rate is about 90% for early COAD patients, but is < 10% for advanced COAD patients [3]. Although the American Joint Committee on Cancer (AJCC) tumor, lymph node, metastases (TNM) staging system has contributed to the treatment of COAD patients, it cannot adequately predict prognosis due to the molecular heterogeneity of COAD [4]. Therefore, early diagnostic and prognostic evaluations are important to improve the treatment and overall survival (OS) of COAD patients.

Epigenetic modifications such as DNA methylation, genomic imprinting and RNA editing alter multiple signaling pathways during the development and progression of COAD [5]. The accumulation of aberrantly methylated DNA sites in intestinal epithelial cells is known to promote the occurrence of COAD [6]. DNA methylation is an important regulator of gene expression [7], and the DNA methylation status has been found to be more reliable than gene expression for the diagnosis of certain cancers [8]. DNA methylation analysis has several advantages, including a high clinical sensitivity and dynamic range, and may provide more dependable markers of COAD than gene mutation analysis [9].

Despite the benefits of DNA methylation analysis, there are limitations to the existing studies. In terms of the genome-wide DNA methylation level, non-CpG-island regions including ‘Open sea,’ ‘Shore’ and ‘Shelf’ regions account for a large proportion of total methylated positions and thus are quite likely to have important effects [10]; however, most studies have focused on abnormal DNA methylation levels in CpG islands in promoter regions. Moreover, in previous studies, methylated diagnostic biomarkers of COAD have not been able to distinguish COAD accurately and consistently from common cancers such as bladder cancer (BLCA), breast cancer (BRCA), cervical cancer (CESC), etc. For example, Sobhani et al. reported that the promoters of certain genes (including *SFRP1*, *2*, *3*, *PENK*, etc.) were hypermethylated in COAD, and Beggs et al. reported that five marker groups (*SFRP2*, *SFRP4*, *WIF1*, *APC1A* and *APC2*) could detect COAD precancerous lesions with modest predictive power (area under the curve [AUC] = 0.83), but the models in these studies could not precisely distinguish COAD from other cancers [11, 12]. Therefore, there is an urgent need for a combined diagnostic model with this ability.

Previous studies have examined not only diagnostic biomarkers, but also prognostic biomarkers of COAD. One feature of a good prognostic biomarker is its independence from clinicopathological prognostic factors. Clinicopathological characteristics such as age [13], gender [14], race [15], AJCC stage [16], examined lymph node count [17] and lymphatic invasion [18] have been identified as the primary predictors of prognosis in COAD. However, studies of methylated prognostic biomarkers thus far have not produced combined prognostic models based on genome-wide CpG sites that can effectively predict the OS of COAD patients independently of these important clinicopathological characteristics. Lind et al. reported that patients with greater methylation of a COAD biomarker group had a worse prognosis, although the difference was not dramatic in multivariate analysis [19]. Liang et al. found that methylation-regulated differentially expressed genes (5 upregulated and 81 downregulated genes) were associated with OS, but the authors did not construct a combined model to systematically predict COAD prognosis [20]. Ahn et al. demonstrated that genes such as *WNT5A*, *SFRP1* and *SFRP2* were prognostic indicators of the high CpG island methylator phenotype in COAD; however, cancer recurrence could only be predicted in resected stage III proximal COAD, not in distal COAD [21]. Thus, there is also a great need for a combined prognostic model that can accurately predict the OS of COAD patients independently of clinicopathological parameters.

A differentially methylated CpG site (DMP) is a CpG site with significantly different mean methylation levels in different groups (e.g., cancer versus normal) [22]. In this study, we used conditional screening and machine learning to obtain DMPs that could be used as specific diagnostic biomarkers for COAD. Then, we constructed and validated a COAD-specific diagnostic model using these DMPs, and evaluated its ability to distinguish COAD from normal tissues and other cancers. Finally, we constructed a combined COAD prognostic model based on six CpG sites, and verified that it could accurately predict high-risk and low-risk COAD patients independently of important clinicopathological parameters.

## RESULTS

### Genomic distribution of hypermethylated and hypomethylated DMPs

**Figure 1:**
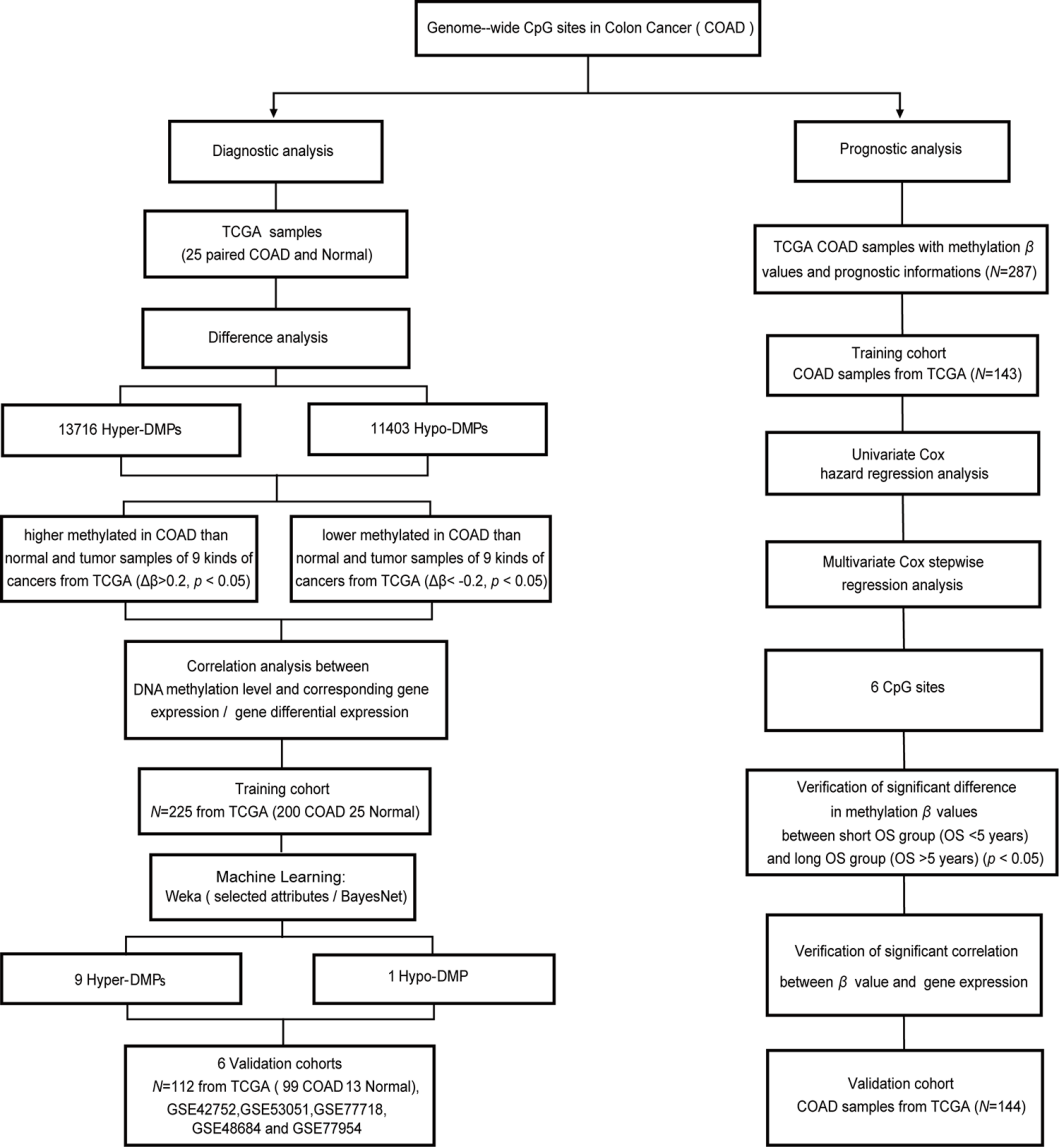
**Workflow diagram for biomarker screening and model construction.** The DNA methylation levels of genome-wide CpG sites were used to screen biomarkers and construct diagnostic and prognostic models of COAD. Left side: diagnostic biomarker selection and COAD-specific diagnostic model construction. Conditional screening and machine learning using the selected attributes and BayesNet functions of WEKA were performed to obtain the final nine Hyper-DMPs and one Hypo-DMP as potential biomarkers in the training cohort from TCGA (including 200 COAD and 25 normal samples). BayesNet was used to evaluate the COAD-specific diagnostic model based on these DMPs in the validation cohort from TCGA (including 99 COAD and 13 normal samples) and five independent GEO cohorts (GSE42752, GSE53051, GSE77718, GSE48684 and GSE77954). Right side: prognostic biomarker selection and COAD prognostic model construction. Univariate Cox hazard regression analysis and multivariate Cox stepwise regression analysis were applied to 143 TCGA COAD samples as the training cohort to obtain six CpG sites as potential biomarkers. The prognostic model based on these six CpG sites was evaluated using 144 TCGA COAD samples as the validation cohort.

**Figure 2:**
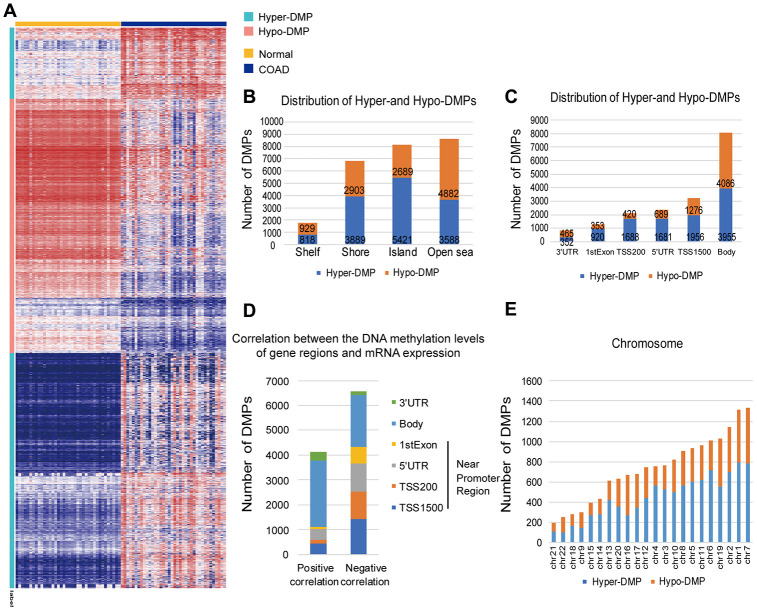
**Distribution of DMPs.** (**A**) Unsupervised hierarchical clustering and heat map display of the methylation levels of the Hyper- and Hypo-DMPs in 25 paired COAD and normal samples from TCGA. (**B**) The distribution of Hyper-DMPs and Hypo-DMPs in different genomic region types. *Island*, a CpG site located within a CpG island; *Shore*, a CpG site located < 2 kilobases from a CpG island; *Shelf*, a CpG site located > 2 kilobases from a CpG island; *Open sea*, a CpG site not in an island or annotated gene. (**C**) The numbers and ratios of Hyper-DMPs and Hypo-DMPs according to their distance from the promoter. *TSS1500*, 200-1500 base pairs upstream of the transcription start site; *TSS200*, 200 base pairs upstream of the transcription start site; *5′UTR*, 5′ untranslated region; *1^st^ Exon*, exon 1; *3′UTR*, 3′ untranslated region. (**D**) The positional distribution (in terms of promoter distance) of the DMPs in which the methylation level correlated positively or negatively with the expression of the corresponding gene (FDR < 0.05). (**E**) Chromosome distribution of Hyper-DMPs and Hypo-DMPs. *Chr*: chromosome.

To explore the abnormal methylation status of the entire genome, we conducted in-depth studies on the early diagnosis and prognostic evaluation of COAD patients (Figure 1). First, we performed CpG site expression profiling analysis between COAD tumor samples (*N* = 25) and paired normal samples (*N* = 25) from The Cancer Genome Atlas (TCGA) cohort. Then, 13716 Hyper-DMPs and 11403 Hypo-DMPs were obtained in these included cohorts. Specifically, unsupervised cluster analysis distinguished these Hyper-DMPs and Hypo-DMPs in 25 paired COAD and normal samples from TCGA (Figure 2A). When we assessed the locations of these Hyper-DMPs and Hypo-DMPs among genomic region types, we observed that Hyper-DMPs were most abundant in Island regions (39%), whereas Hypo-DMPs were mainly distributed in Open sea regions (42%) (Figure 2B). We also determined the enrichment of the DMPs by calculating the ratio of Hyper-DMPs to Hypo-DMPs in each region. The results indicated that Hyper-DMPs were enriched in Island regions (66%; Hyper/Hypo = 5421/2689), whereas Hypo-DMPs occurred more frequently in Open sea regions (58%; Hypo/Hyper = 4882/3588).

More importantly, Hyper-DMPs were mainly located near promoter regions, including TSS1500 (the region 200 to 1500 nucleotides upstream of the transcription start site), TSS200 (the region from the transcription start site to 200 nucleotides upstream of the transcription start site), the 5′ untranslated region (UTR) and the 1^st^ Exon (Figure 2C). However, Hypo-DMPs were mostly enriched in the Body and the 3′UTR, which occupied a large percentage of the regions, genome-wide. The DMP distribution ratio also indicated that proximal promoter regions were mainly hypermethylated (69%; hyper/hypo = 6245/2738), while the proportions of Hyper- and Hypo-DMPs in the Body and 3′UTR were almost equal (51%; hypo/hyper = 4551/4307). Notably, both Hyper-DMPs and Hypo-DMPs occupied a large proportion of the whole genome, about 3.42%.

Next, we calculated Pearson correlation coefficients to determine the correlation between the DNA methylation of the DMPs and the expression of their corresponding genes (Figure 2D). Among the 17112 DMPs for which both the DNA methylation levels and the corresponding mRNA expression profiles were available, the methylation levels of 6565 Hyper-DMPs and 4112 Hypo-DMPs were significantly associated with the mRNA levels of the corresponding genes (|*r*| > 0.1, false discovery rate [FDR] < 0.05). When we analyzed the distance between these DMPs and promoter regions, we found that DMPs in or near promoter regions (i.e., in the 1^st^ Exon, 5′ UTR, TSS200 or TSS1500) were negatively associated with mRNA expression, whereas those outside promoter regions (i.e., in the Body or 3′ UTR) were positively associated with gene expression. Moreover, the DMPs had a higher distribution frequency on chromosomes 7 and 1 than on the other autosomes (Figure 2E). Since the DMPs that significantly altered the expression of their corresponding genes were not limited to promoter regions, we screened the whole genome for potential biomarkers of COAD and constructed a diagnostic prediction model based on the genome-wide Hyper- and Hypo-DMPs.

### Identification of COAD-specific methylation biomarkers and construction of a COAD-specific diagnostic model

Next, in order to construct a diagnostic model to distinguish COAD tumor tissues from normal intestinal epithelial tissues and the tumor tissues of nine other cancer types (BLCA, BRCA, CESC, glioblastoma [GBM], head and neck cancer [HNSC], liver cancer [LIHC], lung adenocarcinoma [LUAD], lung squamous cell carcinoma [LUSC] and endometrial cancer [UCEC]), we performed conditional screening and machine learning studies based on the genome-wide DMPs obtained from TCGA above (13716 Hyper-DMPs and 11403 Hypo-DMPs). For the conditional screening, we determined the average *β* values (a measure of CpG site methylation) of these Hyper-/Hypo-DMPs in all the samples for the nine cancer types in TCGA. Then, we selected DMPs based on an average methylation level difference of at least 0.2 units in COAD. After screening under the above conditions, we obtained 17 Hyper-DMPs and 8 Hypo-DMPs as candidate biomarkers. Further analysis revealed that these 17 Hyper-DMPs and 8 Hypo-DMPs were among the 6565 Hyper-DMPs and 4112 Hypo-DMPs that were significantly associated with the expression of their corresponding genes (log2 |fold change| > 1, FDR < 0.05).

For machine learning, two-thirds of the total tumor and normal samples from the COAD cohort of TCGA (200 COAD and 25 normal samples) were randomly set as the training cohort, while the remaining one-third of the total samples (99 COAD and 13 normal samples) were used as the validation cohort. The *β* values of the 17 Hyper-DMPs and 8 Hypo-DMPs in the training cohort were input into WEKA, and the selected attributes function of WEKA was used to filter these candidate biomarkers. As potential diagnostic biomarkers, nine Hyper-DMPs (cg26036626, cg03882585, cg08130988, cg16733654, cg12587766, cg08808128, cg13004587, cg05038216 and cg09746736) and one Hypo-DMP (cg26718707) were selected to construct a COAD-specific diagnostic model (Table 1). Finally, based on the nine Hyper-DMPs and one Hypo-DMP, we constructed a COAD-specific diagnostic model with BayesNet [23].

**Table 1:** Characteristics of 9 Hyper-DMPs and 1 Hypo-DMP in the COAD-specific diagnostic model.

**Biomarkers**	**Ref Gene**	**Chromosome**	**Start**	**End**	**CGI Coordinate**	**Feature**	**CGI**	**FDR**	**Type**
cg26036626	FBLIM1	chr1	15759102	15759103	15758576-15759367	5'UTR	Island	1.79e-15	Hyper-DMP
cg03882585	SYNE1	chr6	152636775	152636776	152636675-152637337	5'UTR	Island	1.29e-05	Hyper-DMP
cg08130988	EFEMP1	chr2	55923790	55923791	55923205-55923813	1^st^ Exon	Island	6.49e-05	Hyper-DMP
cg16733654	PTPRS	chr19	5293072	5293073	5292760-5294200	5'UTR	Island	7.65e-09	Hyper-DMP
cg12587766	LIFR	chr5	38556333	38556334	38556120-38557461	1^st^ Exon	Island	2.19e-11	Hyper-DMP
cg08808128	CLIP4	chr2	29115566	29115567	29115117-29116043	1^st^ Exon	Island	4.48e-09	Hyper-DMP
cg13004587	SCGB3A1	chr5	180590349	180590350	180590099-180592062	Body	Island	0.0448	Hyper-DMP
cg05038216	CLIP4	chr2	29116225	29116226	29115117-29116043	5'UTR	Shore	1.82e-09	Hyper-DMP
cg09746736	SLC6A2	chr16	55656218	55656219	55655686-55656983	TSS 1500	Island	2.38e-08	Hyper-DMP
cg26718707	DIP2C	chr10	472430	472431	472252-472531	Body	Island	0.0107	Hypo-DMP

CGI: CpG island

**Figure 3:**
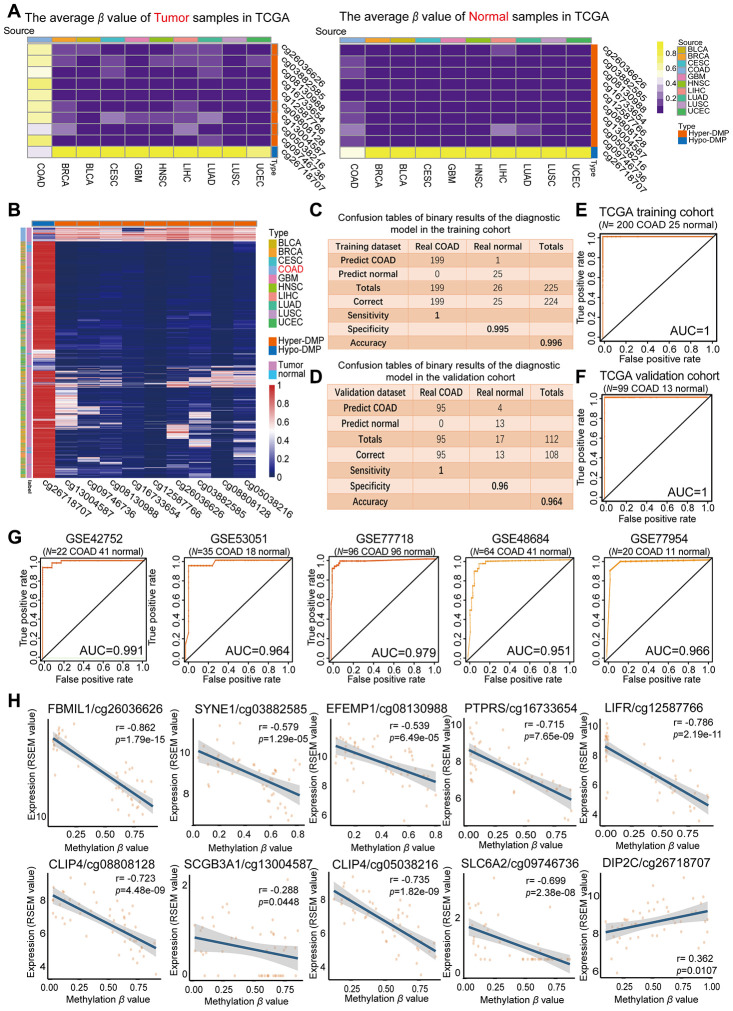
**Evaluation of the COAD-specific diagnostic biomarkers and diagnostic model.** (**A**) Heat maps of the average methylation levels of the nine Hyper-DMPs and one Hypo-DMP in all the samples from 10 cancer types. The legend on the right marks the source and CpG type. The picture on the left represents the tumor samples in TCGA, while the picture on the right represents the normal samples in TCGA. (**B**) Unsupervised hierarchical clustering of the methylation levels of the nine Hyper-DMPs and one Hypo-DMP in all the samples from 10 cancer types. The legend on the right marks the source and CpG type. (**C**–**F**) Confusion tables (**C**, **E**) and corresponding ROC curves (**D**, **F**) for the binary results of the COAD-specific diagnostic model in the training cohort (*N* = 225) and the validation cohort (*N* = 112) from TCGA. (**G**) ROC curves of the COAD-specific diagnostic model in five GEO COAD validation cohorts (GSE42752, GSE53051, GSE77718, GSE48684 and GSE77954, which included 22 COAD and 41 normal samples, 35 COAD and 18 normal samples, 96 paired COAD and normal samples, 64 COAD and 41 normal samples, and 20 COAD and 11 normal samples, respectively). (**H**) The correlation between the DMP methylation level and the expression of the corresponding gene for each diagnostic biomarker, determined through Pearson correlation tests (*r* > 0.2, FDR < 0.05). Gene expression is presented as the RSEM normalized count converted by log2 (*x* + 1).

The average *β* values of the nine Hyper-DMPs and one Hypo-DMP selected for our diagnostic model in all the COAD tissues, normal tissues and nine types of cancerous tissues from TCGA are visualized in Figure 3A. We performed an unsupervised cluster analysis to evaluate these *β* values (Figure 3B), and found that they were clearly divided into four clusters. The COAD tumor samples were significantly differentiated from all the normal samples and the tumor samples from the nine other cancer types based on the nine Hyper-DMPs and one Hypo-DMP.

Subsequently, we used the COAD-specific diagnostic model to train the training cohort (including 200 COAD and 25 normal samples from TCGA) in WEKA. Using BayesNet, we determined that the COAD-specific diagnostic model had a sensitivity of 100%, specificity of 99.5% and accuracy of 99.6% in the training cohort (Figure 3C). In the validation cohort (99 COAD and 13 normal samples from TCGA), the diagnostic model had a sensitivity of 100%, specificity of 96% and accuracy of 96.4% (Figure 3D). Therefore, our COAD-specific diagnostic model was confirmed to perfectly distinguish between COAD and normal samples in the training cohort (AUC = 1) (Figure 3E) and the validation cohort (AUC = 1) from TCGA (Figure 3F).

To demonstrate the versatility of our diagnostic model, we conducted a population heterogeneity analysis using the validation cohort from TCGA, which included samples from 4 Asian, 25 black or African American and 73 white patients. The sensitivity, specificity and accuracy are shown in Table 2. Our diagnostic model exhibited no significant population heterogeneity, suggesting that it can be applied to people of different races. In addition, we used the five independent GEO COAD cohorts mentioned above (GSE42752, GSE53051, GSE77718, GSE48684 and GSE77954) as validation cohorts. In receiver operating characteristic (ROC) analyses, the AUCs of these five cohorts were 0.991, 0.964, 0.979, 0.951 and 0.966, respectively (Figure 3G). These results further illustrated the reproducibility and stability of our COAD-specific diagnostic model.

**Table 2:** The stratification analysis of the sensitivity, specificity, and accuracy from different races including 4 Asian, 25 Black or African American, and 73 White upon TCGA validation cohort.

**Race**	**Asian**	**Black or African American**	**White**
Sensitivity	0	1	1
Specificity	1	0.913	0.97
Accuracy	1	0.92	0.973

We also analyzed the correlation between the DMP methylation level and the expression of the corresponding genes for the nine Hyper-DMPs and the one Hypo-DMP in our diagnostic model. The Hypo-DMP (cg26718707) corresponded to *DIP2C*, while the nine Hyper-DMPs corresponded to eight genes: *FBLIM1* (cg26036626), *SYNE1* (cg03882585), *EFEMP1* (cg08130988), *PTPRS* (cg16733654), *LIFR* (cg12587766), *CLIP4* (cg08808128 and cg05038216), *SCGB3A1* (cg13004587) and *SLC6A2* (cg09746736). The results of the correlation analysis are shown in Figure 3H and Supplementary Table 1. The expression of *DIP2C* correlated positively with the methylation level of the Hypo-DMP (r > 0.1, FDR < 0.05), and the expression of the other eight genes correlated negatively with the methylation levels of the corresponding nine Hyper-DMPs (r < -0.1, FDR < 0.05).

**Figure 4:**
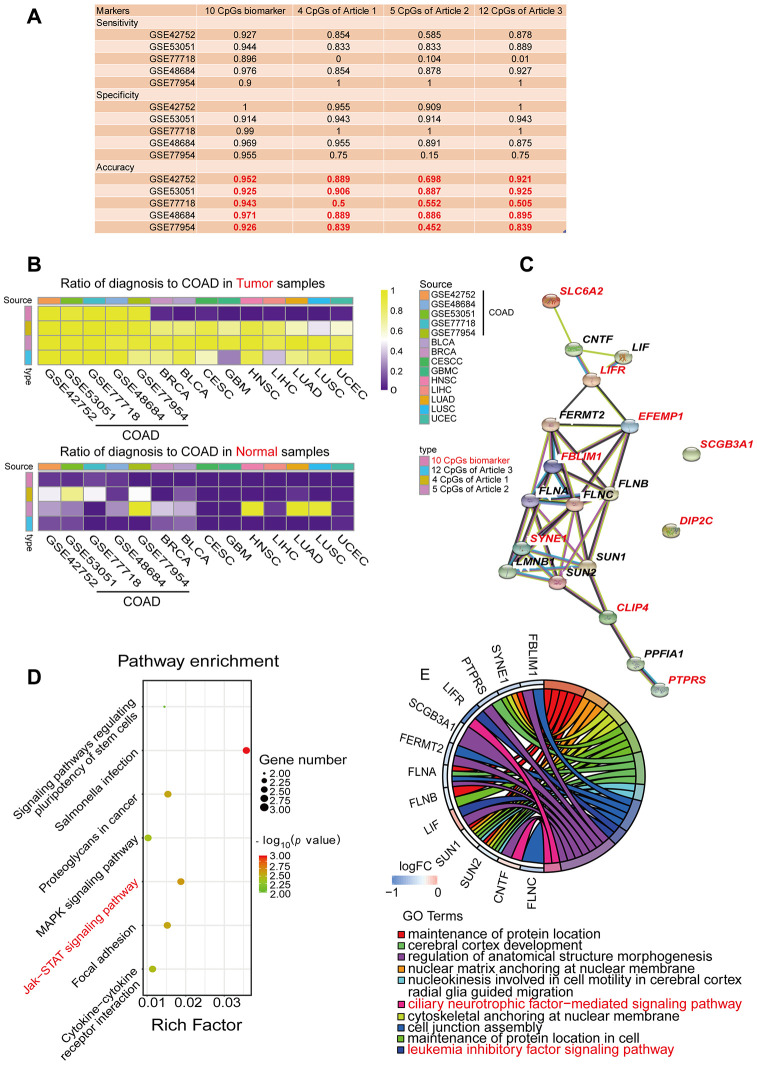
**Performance comparison of diagnostic models and enrichment analysis of the corresponding genes.** (**A**) Table displaying the classification performance of different methylation models for COAD and normal tissues in five independent GEO cohorts (GSE42752, GSE53051, GSE77718, GSE48684 and GSE77954). In addition, Azuara et al. [24] (Article 1) reported four CpG sites as diagnostic biomarkers for COAD, and the methylation values for each of them were available in the COAD cohort from TCGA; Beggs et al. [25] (Article 2) reported six CpG sites as diagnostic biomarkers for COAD, and the methylation values for five of them were available in the COAD cohort from TCGA; and Naumov et al. [26] (Article 3) reported 14 CpG sites as diagnostic biomarkers for COAD, and the methylation values for 12 of them were available in the COAD cohort from TCGA. (**B**) Heat map comparing our diagnostic model with the previous methylation models. Rows are labeled with the different sources of methylation data. The legend indicates that the range is 0-1. The color represents the percentage of the total samples predicted to be COAD. In the cohorts for the nine different cancer types, the ideal results should be 0. (**C**) Predicted protein interaction network of the genes corresponding to the COAD-specific diagnostic biomarkers. Version 11.0 of the STRING protein database was used. The different line colors represent different kinds of correlations between the proteins corresponding to the model (dark blue for coexistence, black for co-expression, pink for an experiment, light blue for a database, green for text mining, and purple for homology). The red genes are the corresponding genes of the diagnostic biomarkers. Note that *CLIP4* is the corresponding gene for both cg08808128 and cg05038216. (**D**, **E**) KEGG (**D**) and GO (**E**) enrichment analysis results from the STRING protein database. All seven results are shown for the KEGG enrichment analysis, and the top 10 results are shown for the GO enrichment analysis, with *p*-values arranged from large to small. In the KEGG enrichment graph (**D**), the X-axis represents the Rich factor, indicating the degree of enrichment (Rich factor = observed gene counts/background gene counts), and the Y-axis represents the enriched KEGG terms. The color represents the -log10 (*p*-value), and the size of the dot represents the number of genes. In the GO enrichment graph (**E**), the GO term indicates the GO enrichment pathway.

Next, using the five independent GEO cohorts, we compared our COAD-specific diagnostic model with three previously reported methylation-based diagnostic models: a Bayesian model including four CpG sites from Azuara et al. [24], a logistic regression model including five CpG sites from Beggs et al. [25] and a logistic regression model including 12 CpG sites from Naumov et al. [26] (Figure 4A). As expected, our model exhibited better sensitivity, specificity and accuracy than the three previously reported diagnostic models in most cases for the five GEO COAD cohorts. We also compared our diagnostic model with these three models in terms of its ability to distinguish COAD from normal tissues and nine other types of cancerous tissues. For this purpose, we divided all the samples from the five GEO COAD cohorts and nine various TCGA cancerous cohorts into a tumor group and a normal group, and we calculated the proportion of samples that were predicted to be COAD among all the samples (Figure 4B, Table 3). In the cohorts from the nine different types of cancers, the ideal proportion would have been 0. When we tested our COAD-specific diagnostic model, almost none of the normal intestinal epithelial samples or the tumor tissues from the nine other cancer types were predicted as COAD (0-5%, median: 0%). However, when we tested the three previously reported diagnostic models, 0-97.7% of the normal tissues (median: 0%, 33.3% and 0%, respectively) and 20.2-98.7% of the tumor tissues from the nine other cancer types (median: 45.4%, 93.5% and 75.2%, respectively) were predicted as COAD. Therefore, our COAD-specific diagnostic model based on nine Hyper-DMPs and one Hypo-DMP not only distinguished COAD from normal samples, but also compensated for the deficiencies of previous COAD diagnostic models that could not differentiate COAD from nine other cancer types.

**Table 3:** Comparison of the performance of different methylation models in all samples of 9 kinds of cancers.

**Accuracy**	**BLCAC**	**BLCAN**	**BRCAC**	**BRCAN**	**CESCC**	**CESCN**	**GBMC**	**GBMN**	**HNSCC**	**HNSCN**
10 DMPs biomarker	0.022	0	0.05	0	0.05	0	0	0	0.02	0
4 CpGs of Article 1	0.593	0.095	0.584	0.01	0.663	0	0.327	0	0.742	0.02
5 CpGs of Article 2	0.939	0.333	0.92	0.388	0.977	0	0.928	0	0.977	0.977
12 CpGs of Article 3	0.872	0.19	0.749	0.143	0.625	0	0.242	0	0.87	0.02
**Accuracy**	**LIHCC**	**LIHCN**	**LUADC**	**LUADN**	**LUSCC**	**LUSCN**	**UCECC**	**UCECN**		
10 DMPs biomarker	0.03	0	0.01	0	0	0	0.04	0		
4 CpGs of Article 1	0.413	0	0.454	0	0.202	0	0.348	0		
5 CpGs of Article 2	0.871	0.08	0.987	1	0.935	0.93	0.843	0.043		
12 CpGs of Article 3	0.375	0	0.752	0	0.815	0	0.944	0.043		

The vertical axis shows the different sources of the methylation models. The horizontal axis shows the tumor and normal methylation cohorts from 9 kinds of cancer. Affix C represents the tumor samples of the methylation data sets. Affix N represents the normal sample of the methylation cohorts. The numbers represent the percentage of samples predicted to be COAD in the total samples. The cohorts in the table are from 9 kinds of cancers, and the ideal result should be 0. Azuara D et al. [24] (Article 1)reported 4 CpG sites as diagnostic markers for COAD, and 4 of them had methylation values in the TCGA COAD cohort; Beggs AD et al. [25] (Article 2)report 6 CpG sites as diagnostic markers for COAD, and 5 of them had methylation values in the TCGA COAD cohort; Naumov VA et al. [26] (Article 3)reported 14 CpG sites as diagnostic markers for COAD, and 12 of them had methylation values in the TCGA COAD cohort.

Then, we used the STRING database to construct a protein-protein interaction network for the nine genes corresponding to the nine Hyper-DMPs and the one Hypo-DMP (Figure 4C). We also performed Gene Ontology (GO) and Kyoto Encyclopedia of Genes and Genomes (KEGG) pathway enrichment analyses on these genes (Figure 4D and 4E). We found that the nine genes were involved in important signaling pathways of tumorigenesis and development, such as Salmonella infection, Janus kinase (JAK)/signal transducer and activator of transcription (STAT) signaling, focal adhesion, proteoglycans in cancer, cytokine-cytokine receptor interactions, etc. All the results of the KEGG pathway analysis and the top 10 results of the GO analysis are shown in Supplementary Tables 2 and 3.

The above results demonstrated that our COAD-specific diagnostic model could accurately and precisely distinguish COAD tissues from normal intestinal epithelial samples and tumor samples from nine cancer types, and that the nine Hyper-DMPs and one Hypo-DMP included in this model may be potential biomarkers for the early prediction and specific diagnosis of COAD.

### Identification of prognostic biomarkers of COAD and construction of a combined COAD prognostic model

**Figure 5:**
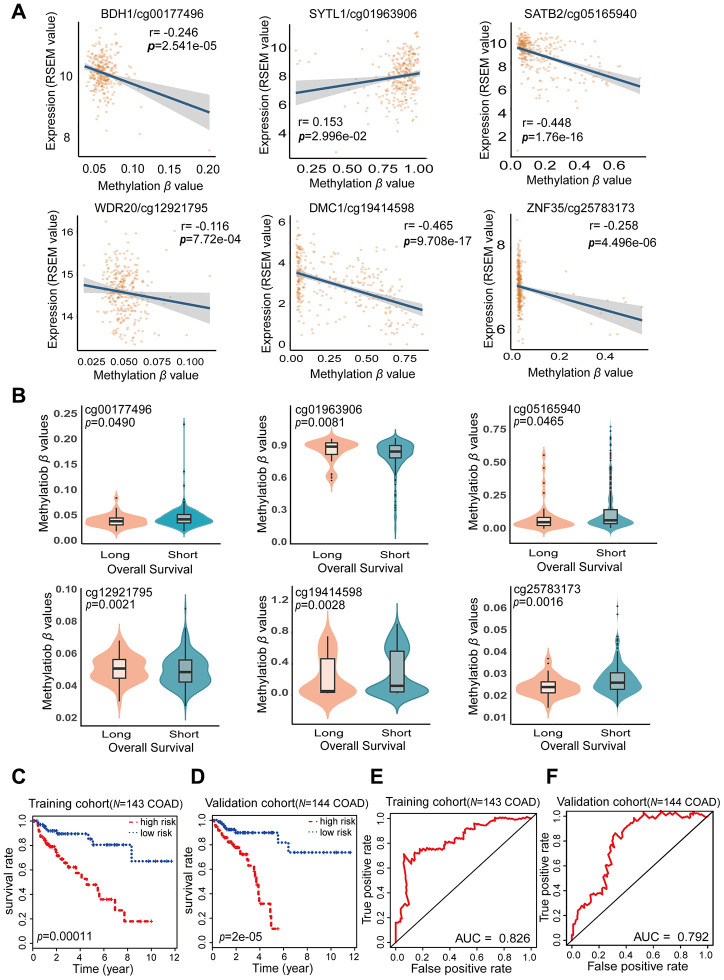
**Characteristics of the potential prognostic biomarkers and evaluation of the combined prognostic model based on six CpG sites.** (**A**) The correlations between the methylation β levels of the prognostic biomarkers and the expression of the corresponding genes were evaluated with Pearson correlation tests. Gene expression is presented as the RSEM normalized count converted by log2 (x + 1). (**B**) Violin plots of the methylation β values for patients with longer (> 5 years) and shorter (< 5 years) OS in the training cohort, with the median in the centerline. A Wilcoxon test was used to determine the difference between the two groups. The corresponding CpG sites, cor-values and p-values are shown at the top of the plot. (**C**, **D**) Kaplan-Meier analysis was performed on the OS of high-risk and low-risk patients using our prognostic model in the training (N = 143) (**C**) and validation (N = 144) (**D**) cohorts from TCGA. The difference in OS between the two groups was determined with a log-rank test. Higher risk scores were associated with significantly poorer OS. Patients were divided into low-risk and high-risk groups using the median risk score as the cut-off. (**E**, **F**) ROC curves showing the sensitivity and specificity of the prognostic model in predicting patients’ OS in the training (N = 143) (**E**) and validation (N = 144) (**F**) cohorts from TCGA.

A total of 287 COAD tissue samples in the cohort from TCGA had both methylated *β* values and corresponding prognostic information. The distribution and corresponding demographic characteristics of these patients are summarized in Table 4. The patients were divided into a training cohort (*N* = 143) and a validation cohort (*N* = 144). The training cohort was used to obtain prognostic biomarkers and construct a COAD prognostic model, while the validation cohort was used to test the COAD prognostic model. Univariate Cox hazard regression analysis of the training cohort revealed 64 CpG sites that correlated significantly with the OS of COAD patients (FDR < 0.05); thus, these CpG sites were identified as candidate prognostic biomarkers. Multivariate Cox stepwise regression analysis was applied to these 64 CpG sites, and six sites (cg00177496, cg01963906, cg05165940, cg12921795, cg19414598 and cg25783173) were included in our final hazard ratio model, which was constructed as a combined COAD prognostic model for OS prediction (Table 5). The six CpG sites from our COAD prognostic model were found to correspond to *BDH1* (cg00177496), *SYTL1* (cg01963906), *SATB2* (cg05165940), *WDR20* (cg12921795), *DMC1* (cg19414598) and *ZNF35* (cg25783173) (Figure 5A and Supplementary Table 4). The expression of *SYTL1* correlated positively with the methylation level of cg01963906 (r > 0.1, FDR < 0.05), and the expression of the other five genes correlated negatively with the methylation levels of the remaining CpG sites (r < -0.1, FDR < 0.05).

**Table 4:** Clinicopathological characteristics of COAD patients from the TCGA database.

**Characteristics**	**Patients**					
	**Total (*N* = 287)**		**Training cohort (*N* = 143)**		**Validation cohort (*N* = 144)**	
	**No**	**%**	**No**	**%**	**No**	**%**
***Age***						
≤64	130	45.30	58	40.56	72	50
>64	157	54.70	85	59.44	72	50
***Histological type***						
Colon Adenocarcinoma	246	85.71	119	83.22	127	88.19
Colon Mucinous Adenocarcinoma	38	13.24	22	15.38	16	11.11
Unknown	3	1.05	2	1.40	1	0.70
***Pathologic M***						
M0	195	67.94	102	71.33	93	64.58
M1	40	13.94	21	14.69	19	13.19
MX	49	17.07	19	13.29	30	20.83
Unknown	3	1.05	1	0.07	2	1.39
***Pathologic N***						
N0	166	57.84	73	51.05	93	64.58
N1	73	25.44	46	32.17	27	18.75
N2	48	16.72	24	1.40	24	16.67
***Pathologic T***						
T1	7	2.44	2	1.39	5	3.47
T2	42	14.63	17	11.89	25	17.36
T3	199	69.34	105	73.43	94	65.28
T4	38	13.24	19	13.29	19	13.19
Unknown	1	0.35	0	0	1	0.70
***Gender***						
Female	134	46.69	70	48.95	64	44.44
Male	153	53.31	73	51.05	80	55.56
***Race***						
American Indian or Alaska Native	1	0.35	1	0.70	0	0
Asian	11	3.83	4	2.80	7	4.86
Black or African American	57	19.86	20	13.98	37	25.70
White	201	70.04	108	75.52	93	64.58
Unknown	17	5.92	10	7.00	7	4.86
***Tumor stage***						
Stage I	43	14.98	16	11.19	27	18.75
Stage II	110	38.33	52	36.36	58	40.28
Stage III	84	29.27	51	14.69	33	22.92
Stage IV	40	13.94	21	2.10	19	13.19
Unknown	10	3.48	3	27.27	7	4.86
***Lymphatic invasion***						
Yes	76	26.48	39	27.27	37	25.69
No	175	60.98	88	61.54	87	60.42
Unknown	36	12.54	16	11.19	20	13.89
***Primary lymph node presentation assessment***						
Yes	265	92.33	134	93.70	131	90.97
No	14	4.88	5	3.50	9	6.25
Unknown	8	2.79	4	2.80	4	2.78
***Vital status***						
Alive	218	75.96	107	74.83	111	77.08
Dead	69	24.04	36	25.17	33	22.92
***Longest dimension***						
≥2	43	14.98	27	18.88	16	11.11
<2	175	60.98	90	62.94	85	59.03
Unknown	69	24.04	26	18.18	43	29.86
***Sample type***						
Metastatic	1	0.35	0	0	1	0.69
Primary Tumor	285	99.30	142	99.30	143	99.31
Recurrent Tumor	1	0.35	1	0.70	0	0
***Lymph node examined count***						
≥12	226	78.75	117	81.82	109	75.70
<12	42	14.63	19	13.28	23	15.97
Unknown	19	6.62	7	4.90	12	8.33

**Table 5:** Characteristics of prognostic biomarkers and their coefficients in the combined COAD prognostic model.

**Biomarkers**	**Ref Gene**	**Coefficients**	**HR**	**CI (lower)**	**CI (upper)**	**SE**	***z* value**	**CGI**	**FDR**
cg00177496	BDH1	38.52	5.3E+16	3.55E+02	8.02E+30	16.66	2.312	Island	2.541e-05
cg01963906	SYTL1	-4.13	0.01608	1.86E-03	1.39E-01	1.102	-3.75	Island	2.996e-02
cg05165940	SATB2	2.574	13.12	2.70E+00	6.38E+01	0.8072	3.189	Island	1.76e-16
cg12921795	WDR20	-79.32	3.58E-35	2.16E-57	5.91E-13	26.1	-3.039	Island	7.72e-04
cg19414598	DMC1	2.31	10.07	2.75E+00	3.68E+01	0.6616	3.491	Island	9.708e-17
cg25783173	ZNF35	6.061	429	1.60E+01	1.15E+04	1.677	3.614	Island	4.496e-06

HR: Hazard Ratio; CI: 95.0% confidence interval; SE: standard errors of coefficients; z value: Wald z-statistic value.

The risk score formula for our COAD prognostic model was based on the regression coefficients and methylation levels of the six CpG sites, as follows: risk score = (38.52 × cg00177496 *β* value) – (4.13 × cg01963906 *β* value) + (2.574 × cg05165940 *β* value) – (79.32 × cg12921795 *β* value) + (2.31 × cg19414598 *β* value) + (6.061 × cg25783173 *β* value). In the risk score formula, a positive coefficient for a CpG site (cg00177496, cg05165940, cg19414598 and cg25783173) indicates that hypermethylation of that site was associated with shorter OS in COAD patients. In contrast, a negative coefficient for a CpG site (cg01963906 and cg12921795) indicates that greater methylation of that site was associated with longer OS. Our COAD prognostic model revealed that there were significant differences in DNA methylation levels between patients with long-term (> 5 years) and short-term (< 5 years) survival (FDR < 0.05) (Figure 5B). Consistent with the results of the multivariate Cox stepwise regression analysis, the CpG sites with positive coefficients (cg00177496, cg05165940, cg19414598 and cg25783173) exhibited lower methylation levels in patients who survived long-term, while the CpG sites with negative coefficients (cg01963906 and cg12921795) exhibited higher methylation levels in patients who survived long-term. Thus, our combined COAD prognostic model based on six CpG sites successfully distinguished long-term from short-term surviving patients in the training cohort of 143 COAD samples from TCGA.

Based on the Cox regression analyses, risk scores were used as continuous variables in the training and validation cohorts. The risk scores obtained from the combined COAD prognostic model correlated significantly with the OS of COAD patients (Training cohort: likelihood ratio test = 45, *p* < 0.0001; Wald test = 41.93, *p* < 0.0001; score [log-rank] test = 50.34, *p* < 0.0001. Validation cohort: likelihood ratio test = 27.63, *p* < 0.0001; Wald test = 33.48, *p* < 0.0001; score [log-rank] test = 39.33, *p* < 0.0001). Using the median risk score of the training cohort as a cut-off value, we generated Kaplan-Meier curves and performed log-rank tests on the training cohort (Figure 5C) (*p* = 0.00011) and the validation cohort (Figure 5D) (*p* = 2e-05). Through these analyses, we sought to compare the OS of patients in the high-risk and low-risk groups and thus determine the predictive value of the combined COAD prognostic model based on six CpG sites. The risk scores for the training and validation cohorts are shown in Supplementary Tables 5 and 6. The survival rate of patients was significantly greater in the low-risk group than in the high-risk group. These results confirmed that our combined prognostic model based on six CpG sites could classify patients into high-risk and low-risk groups, demonstrating its clinical practicability.

To further evaluate the specificity of our combined COAD prognostic model in predicting survival, we used the AUC values obtained from time-dependent ROC analyses as categorical variables. In both the training cohort and the validation cohort, the combined COAD prognostic model precisely predicted the survival of COAD patients, with AUC values of 0.826 and 0.792, respectively (Figure 5E and 5F). We also performed univariate Cox regression analyses of the six individual CpG sites included in the COAD prognostic model (Supplementary Figure 1). The calculated AUC values indicated that the six individual CpG sites could also distinguish high-risk from low-risk patients; however, the predictive effect of any one CpG site was not as good as the predictive effect of the combined prognostic model using all six CpG sites. These results demonstrated that the six CpG sites may be potential prognostic biomarkers of COAD, but the combined COAD prognostic model based on six CpG sites is more valuable than the individual sites for clinical validation and prognostic evaluation.

### Independence of the combined COAD prognostic model in OS prediction, and comparison of our prognostic model with other known prognostic models

**Figure 6:**
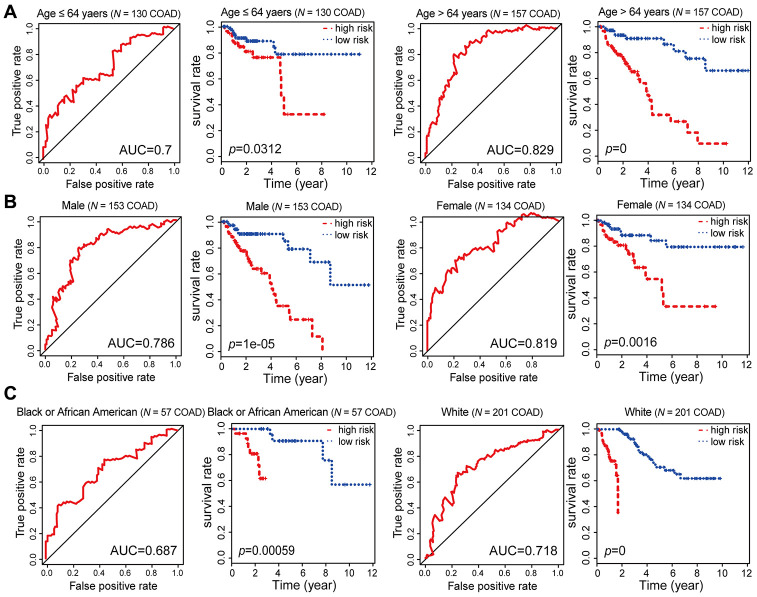
**Kaplan-Meier and ROC analysis results based on age, gender and race.** (**A**) Grouping of COAD patients according to their age at first diagnosis: ≤ 64 years (*N* = 130, 45.30%), > 64 years (*N* = 157, 54.70%). (**B**) Grouping of COAD patients according to gender: male (*N* = 153, 53.31%), female (*N* = 134, 46.69%). (**C**) Grouping of COAD patients according to race: black or African American (*N* = 57, 21.19%), white (*N* = 201, 74.72%).

**Figure 7:**
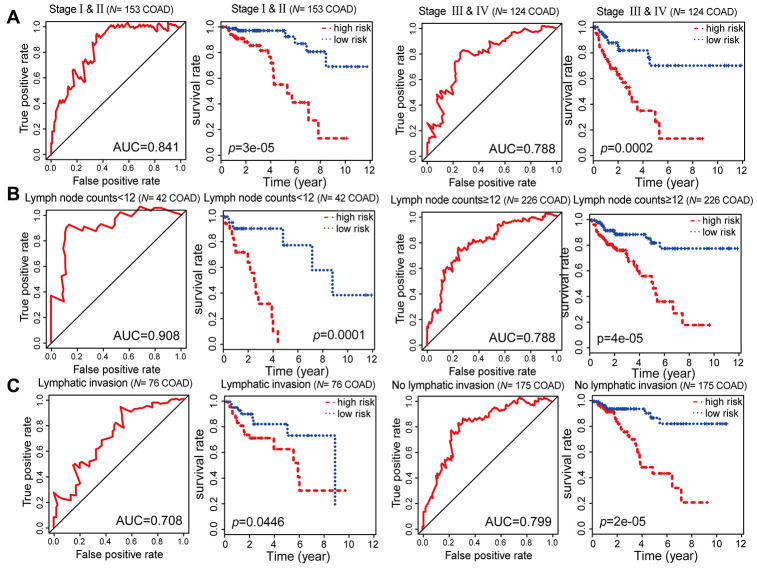
**Kaplan-Meier and ROC analysis results based on stage, examined lymph node count and lymphatic invasion.** (**A**) Grouping of COAD patients according to stage: early (stage I and II [*N* = 153, 53.31%]) and advanced (stage III and IV [*N* = 124, 43.21%]). (**B**) Grouping of COAD patients according to examined lymph node count: < 12 (*N* = 42, 14.63%) and ≥ 12 (*N* = 226, 78.75%). (**C**) Grouping of COAD patients according to lymphatic invasion: lymphatic invasion (*N* = 76, 26.48%) and no lymphatic invasion (*N* = 175, 60.98%).

To evaluate the stability and independence of our combined COAD prognostic model based on six CpG sites, we stratified patients according to clinicopathological characteristics such as age, gender, race, AJCC stage, examined lymph node count and lymphatic invasion. Remarkably, the Kaplan-Meier plots displayed significant extension of OS in the low-risk groups for all these characteristics in the 287 COAD samples from TCGA. Nevertheless, the combined COAD prognostic model predicted the survival of COAD patients more precisely than these factors, with an AUC value of 0.687 (Figure 6A–6C, Figure 7A–7C and Supplementary Figure 2). These results confirmed that the combined COAD prognostic model based on six CpG sites provided an excellent reference for different populations and was an independent predictor of patient survival.

**Figure 8:**
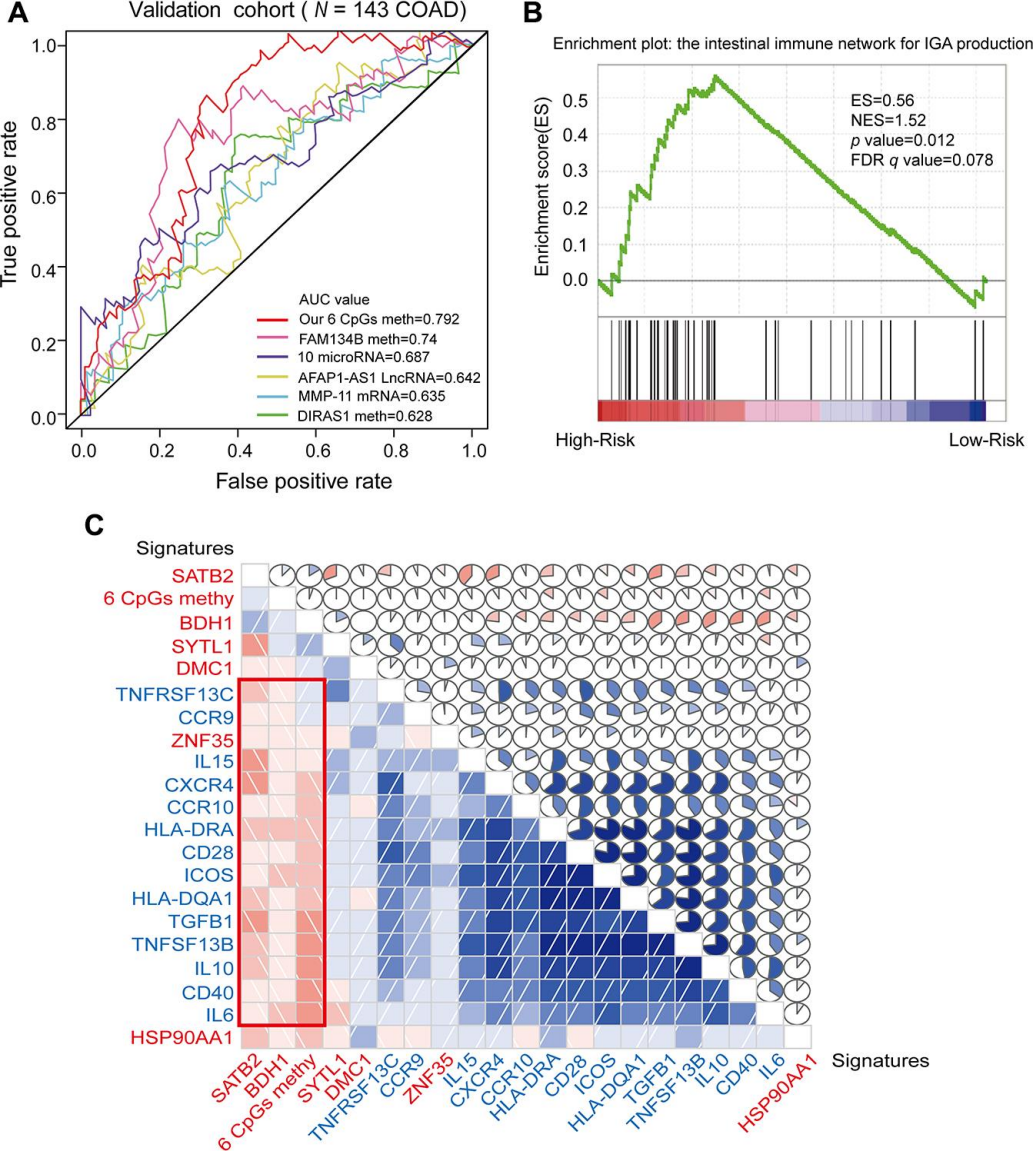
**ROC analysis of different prognostic biomarkers and functional enrichment analysis of the corresponding genes.** (**A**) ROC curve showing the sensitivity and specificity of our prognostic model and other known models in predicting the OS of patients in the validation cohort from TCGA. (**B**) COAD samples were divided into high-risk and low-risk groups, and the enrichment of IINIP pathway gene expression was analyzed using GSEA. *ES*, concentration fraction; *NES*, standardized ES; *p-value*, normalized *p*-value; *FDR q-value*, *p*-value corrected by the FDR method. (**C**) Correlation of the expression of the core enrichment genes from the IINIP pathway, the combined methylation level of our prognostic model and the expression of the genes corresponding to the individual CpG sites of the COAD prognostic biomarkers. The red signature represents the expression of the genes corresponding to the six CpG sites and the six-site combined methylation value; the blue signature represents the expression of the core enrichment genes in the IINIP pathway. Lower triangle: grids showing the correlation between two signatures, where blue indicates a positive correlation and red indicates a negative correlation. Upper triangle: circles represent the one-to-one correlation coefficients, differentiated by the fill area and intensity of shading. Blue indicates a positive correlation and red indicates a negative correlation.

In recent years, DNA methylation biomarkers have been increasingly recognized as important prognostic predictors in COAD. Previously identified markers of COAD prognosis have included hypermethylation of *FAM134B* [27], higher expression of *MMP-11* [28], abnormal methylation and expression of *DIRAS1* [29], upregulation of the long non-coding RNA *BLACAT1* (a cell cycle regulator) [30] and abnormal expression of 10 microRNAs (including hsa-mir-891a, hsa-mir-6854, etc.) [31]. Dai et al. [32] demonstrated that combined biomarkers of DNA methylation were more sensitive and specific than individual DNA methylation markers. To compare the survival prediction ability of our combined prognostic predictive model with those of previously reported biomarkers, we performed ROC analyses of the previous mRNA, long non-coding RNA and microRNA biomarkers in the validation cohort. Our combined COAD prognostic model based on six CpG sites had a much higher AUC value than the other prognostic biomarkers assayed by ROC analysis in the COAD validation cohort from TCGA (*N* = 144) (Figure 8A). These results suggested that our combined COAD prognostic model provided more reliable and sensitive predictions of OS than other biomarkers in COAD patients.

Next, we performed a gene set enrichment analysis (GSEA) on the high- and low-risk groups that had been classified according to the median risk score. The pathways that correlated significantly with risk are illustrated in Figure 8B, Supplementary Figure 3 and Supplementary Table 7 (enrichment score [ES] > 0.4, |normalized enrichment score [NES]| > 1, *p*-value < 0.05 and FDR *q-*value < 0.25). We selected the intestinal immune network for IgA production (IINIP) for further analysis, since this pathway is known to be involved in COAD. The core enrichment genes of the IINIP pathway were obtained via GSEA (Supplementary Table 8). To determine whether the genes corresponding to the combined COAD prognostic biomarkers functioned in IINIP pathways, we conducted a one-to-one correlation analysis on the expression of the core enrichment genes from the IINIP pathway, the combined methylation level of our prognostic model and the expression of the genes corresponding to the individual CpG sites of the COAD prognostic biomarkers (Figure 8C and Supplementary Figure 4). As expected, the expression of *BDH1* and *SATB2* and the combined methylation level of our COAD prognostic model based on six CpG sites correlated significantly with the IINIP signaling pathway (*p* < 0.05). The above results indicated that our combined COAD prognostic model not only accurately predicted prognosis, but also included CpG sites that may directly or indirectly influence the IINIP pathway.

## DISCUSSION

This study was based on a comprehensive Illumina Infinium Human Methylation 450K array dataset in TCGA. To screen for potential diagnostic biomarkers, we first used 25 paired COAD and normal samples from TCGA to obtain Hyper-/Hypo-DMPs. Then, using conditional screening and machine learning based on these genome-wide DMPs, we obtained nine Hyper-DMPs and one Hypo-DMP as the final diagnostic biomarkers for inclusion in our COAD-specific diagnostic model. Our model could accurately and precisely distinguish COAD from normal tissues and nine types of cancerous tissues. Next, to screen for potential prognostic biomarkers, we performed a univariate Cox hazard regression analysis and a multivariate Cox stepwise regression analysis based on genome-wide CpG sites. We identified six CpG sites as potential prognostic biomarkers and used them to construct a combined COAD prognostic model. This model could predict the prognosis of COAD patients independently of important clinicopathological characteristics such as age, gender, race, AJCC stage, examined lymph node count and lymphatic invasion.

Combined DNA methylation models for the diagnosis of COAD have been constructed previously [24–26], and we compared our model with these models. A common problem with most of the previous COAD methylation diagnostic models was that they were only screened and constructed using COAD datasets. Although our diagnostic model was based on data from multiple cancer types, we could not identify DMPs that differentiated COAD from rectal cancer by this modeling method. Colon and rectal tumors were previously considered to differ in their epidemiology and treatment requirements [33]; however, newly published data from TCGA project [4] suggest that the overall patterns of methylation, mRNA and microRNA changes in colon and rectal cancers are indistinguishable. Thus, in future studies, we can try other modeling methods to distinguish COAD from rectal cancer and other cancers of the intestinal system.

We conducted in-depth KEGG and GO pathway enrichment analyses of the genes corresponding to the nine Hyper-DMPs and one Hypo-DMP, and we analyzed their protein-protein interactions in the STRING database. The leukemia inhibitory factor signaling pathway and the ciliary neurotrophic factor-mediated signaling pathway, both of which can activate the JAK2/STAT3 signaling pathway [34, 35], were found to be enriched in our GO analysis. Coincidentally, the JAK/STAT pathway was found to be enriched in our KEGG pathway analysis. JAK/STAT signaling, especially the overactivation of STAT3 and STAT5, is known to promote tumor cell survival, proliferation, and invasion [36]. Therefore, it is significant that our COAD-specific diagnostic biomarkers were both directly and indirectly associated with the JAK/STAT pathway.

The parameters of age, gender, race, AJCC stage, examined lymph node count and lymphatic invasion have been identified as important clinicopathological features of COAD prognosis. Specifically, age was found to be the most important prognostic factor for stage II COAD patients [13], women had a better prognosis than men [14], whites had a higher colorectal cancer survival rate than blacks [15], the AJCC TNM staging system was found to be a necessary adjuvant chemotherapy guide for stage II and III COAD patients [16], the examined lymph node count had excellent prognostic value for COAD patients undergoing surgery [17] and lymphatic invasion diagnosis was found to be an important indicator of lymph node metastasis in T1 COAD [18]. Since early-stage patients are more likely to be cured, prognostic markers that can effectively predict the risk of these patients will have higher clinical value [37]. Importantly, our combined prognostic prediction model based on six CpG sites was independent of these important clinicopathological characteristics of COAD, and had the potential to accurately predict the biological behavior of COAD at an early stage.

The six CpG sites included in our prognostic model were all CpG islands (dense clusters of CpG sites). Abnormal methylation of CpG islands is associated with the silencing of tumor suppressor genes. Two mechanisms have been proposed to explain the transcriptional inhibition caused by CpG island methylation. One proposed mechanism is that CpG islands directly impede the binding of specific transcription factors to recognition sites in promoters [38, 39]. The other proposed mechanism is that proteins that recognize methylated CpG sites, namely methyl CpG binding proteins, stimulate the inhibitory potential of methylated DNA [40].

When we searched the literature for information about the genes corresponding to the six CpG sites in our prognostic model (*BDH1*, *SYTL1*, *SATB2*, *WDR20*, *DMC1* and *ZNF35*), we found that *ZNF35* and *SATB2* have already been established as reliable prognostic marker genes for COAD. For example, *ZNF35* was found to differentiate the prognoses of COAD patients in a validation on independent test sets [41], and the transcription factor *SATB2* was identified as a highly specific marker in colorectal adenocarcinoma when used in conjunction with *CK20* [42]. On the other hand, low expression of *DMC1* has been reported as a poor prognostic marker of ovarian cancer, together with high expression of *XPC* and *RECQL* [43]. These studies indirectly illustrate the reliability of our prognostic model.

Notably, GSEA revealed that our combined COAD prognostic model based on six CpG sites was significantly associated with core enrichment genes of the IINIP pathway, including *HLA-DQB1*, *interleukin (IL)-6*, *IL-15* and *CCR9*. The IINIP pathway has been reported to alter the proliferation of COAD cells, the prognosis of COAD patients, the susceptibility of individuals to COAD, the effectiveness of immunotherapy for COAD, etc. [44–47]. These results suggested that our combined prognostic model could not only predict the prognosis of patients with COAD, but also reflect the immune pathways of COAD. Interestingly, IL-6 has been reported to participate with JAK2/STAT3 in a signaling pathway that promotes COAD cell proliferation [48], and the genes corresponding to the nine Hyper-DMPs and one Hypo-DMP in our COAD-specific diagnostic model were associated with the JAK/STAT pathway.

In summary, by analyzing the genome-wide methylation data of 299 COAD samples and 38 normal samples from TCGA, we found nine Hyper-DMPs and one Hypo-DMP that could be used as potential methylation biomarkers for the diagnosis of COAD. Our COAD-specific diagnostic model based on these DMPs not only differentiated COAD tissues from normal tissues with excellent accuracy and stability, but also precisely distinguished COAD from nine other cancer types (BLCA, BRCA, CESC, GBM, HNSC, LIHC, LUAD, LUSC and UCEC). Furthermore, using 287 COAD samples with prognostic information, we constructed a combined COAD prognostic evaluation model based on six CpG sites. Our model predicted the prognosis of COAD independently of important clinicopathological characteristics such as age, gender, race, AJCC stage, examined lymph node count and lymphatic invasion. Thus, both our COAD-specific diagnostic model and our combined prognostic model have high predictive capabilities and can be applied to the design of adjuvant chemotherapy clinical trials for early COAD patients.

## MATERIALS AND METHODS

### Data source

We downloaded DNA methylation, gene expression and COAD clinical data from TCGA using the University of California Santa Cruz Xena tool (http://xena.ucsc.edu). The DNA methylation data were generated using the Illumina Human Methylation 450 Bead Chip platform, with methylation levels ranging from 0 to 1. We collected the methylation levels of ten types of tumor tissues and normal tissues from TCGA: COAD (299 tumors, 38 normal), BLCA (413 tumors, 21 normal), BRCA (790 tumors, 98 normal), CESC (309 tumors, 3 normal), UCEC (432 tumors, 46 normal), GBM (153 tumors, 2 normal), HNSC (530 tumors, 50 normal), LIHC (379 tumors, 50 normal), LUAD (460 tumors, 32 normal) and LUSC (372 tumors, 43 normal). We calculated the average methylation level of multiple samples as the methylation level of a given CpG site. The level-3 gene expression data were downloaded from RNA-seq HiSeqV2 based on the Illumina HiSeq 2000 RNA sequencing platform, and were obtained as RNA-Seq by Expectation-Maximization (RSEM) [49] normalized counts converted by log2 (*x* + 1). RSEM software was used to normalize counts on the Xena website.

In addition, we downloaded five DNA methylation array cohorts from GEO (https://www.ncbi.nlm.nih.gov/geo/) as independent validation cohorts. (1) The GSE42752 cohort [26] included 22 COAD samples and 41 normal samples from Russia. Quality control was performed using the R package *GenomeStudio* (v. 2011.1). Samples were removed if *p* was > 0.05 and the CpG coverage was < 95%. Thereafter, *GenomeStudio* was used to normalize the DNA methylation data. The methylation levels of the CpG sites were calculated as *β* values, where *β* = intensity (methylated)/intensity (methylated + unmethylated). The data were further normalized using a peak correction algorithm embedded in the *IMA* R package. Finally, CpG sites on sex chromosomes were removed, and the remaining CpG sites were retained for further analysis. (2) The GSE53051 cohort [50] included 35 COAD samples and 18 normal samples from the US. The R package *minfi* was used to preprocess the methylation data. To analyze the methylation levels of CpG sites, we averaged the values of all individuals in normal colon and colon cancer. We compared the data from colon cancer and normal control tissues using *t-*tests. We defined CpG sites with *q* values < 0.05 and a difference magnitude > 0.1 as important CpG sites. (3) The GSE77718 cohort [51] included 96 COAD samples and 96 normal samples from New Zealand. The CpG sites of each sample were preprocessed and rescaled to ensure that the internal control CpG sites had a common mean across samples. CpG sites located on sex chromosomes or known to cross-react with other regions of the genome were excluded from further analyses. The methylation of the remaining CpG sites was corrected using the COMBAT algorithm to account for batch effects (between-array effects). (4) The GSE48684 cohort [52] included 64 COAD samples and 41 normal samples from the US. CpG sites were removed if *p* was > 0.05 in the Illumina Infinium DNA methylation data. Thereafter, the R package *minfi* was used for normalization, including Illumina background level correction, color adjustment and Subset-quantile Within Array Normalization. CpG sites beginning with “rs” on the array were excluded, along with non-CpG sites associated with the X chromosome. The COMBAT algorithm was used to evaluate and correct the batch processing effects of all array runs. (5) The GSE77954 cohort [53] included 20 COAD samples and 11 normal samples from the US. The microarray data were collected at Expression Analysis Inc. (Durham, NC, USA) and preprocessed using the R package *methylamine*. The array platform was the Human Methylation 450 Bead Chip (GPL13534). We normalized the data from each cohort using the R package *limma*.

### Difference and correlation analysis of DNA methylation and corresponding gene expression

Paired samples are the most suitable for assessing differential methylation levels among individuals [54]. Therefore, we used 25 paired samples to obtain DMPs. CpG sites with > 10% missing values were excluded during the screening process. Missing values in the remaining CpG sites were replaced by the median of the cohort. Then, a paired *t*-test was used to obtain DMPs between COAD and normal tissues, and the FDR values were adjusted by the Bonferroni method. CpG sites on the sex chromosomes were removed. CpG sites with |Δ*β*| > 0.2 and FDR values < 0.05 were considered differentially methylated. The R package *Pheatmap* [55] was used for heat mapping and unsupervised cluster analysis.

When a CpG site corresponded to multiple genes, the optimal corresponding gene was obtained using the R package *Champ* [56]. We used TCGA data from 25 paired patients with both COAD and normal expression profiles for differential gene expression analysis. The R package *limma* was used to identify differentially expressed genes from the original data. Genes with a log2 |fold change| > 1 and FDR < 0.05 were considered differentially expressed. Pearson correlation coefficients were calculated to assess the association between DNA methylation and gene expression. Correlations were considered significant based on an |*r* (cor-value)| > 0.1 and FDR < 0.05. All FDR values were adjusted by the Bonferroni method.

### Acquisition of candidate diagnostic biomarkers and construction of a diagnostic model

The 299 COAD and 38 normal samples from TCGA were randomly assigned to the training and validation cohorts at a ratio of 2:1. Five independent GEO cohorts (GSE42752, GSE53051, GSE77718, GSE48684 and GSE77954) were also used as validation cohorts. Firstly, 25 paired COAD and normal samples were compared to obtain DMPs. Secondly, TCGA data from 10 cancer types were used to further screen and narrow the range of candidate DMPs. Candidate Hyper-DMPs were required to have an average methylation level in COAD that was 0.2 units higher than the average methylation level in the normal samples and the nine other types of tumor and normal samples (BLCA, BRCA, CESC, GBM, HNSC, LIHC, LUAD, LUSC and UCEC). Candidate Hypo-DMPs were required to have an average methylation level in COAD that was 0.2 units lower than the average methylation level in the normal samples and the nine other types of tumor and normal samples. Thirdly, we evaluated whether the candidate Hyper-/Hypo-DMPs were significantly associated with the expression of their corresponding genes (|*r*| > 0.1 and FDR < 0.05), and whether the corresponding genes were differentially expressed genes (log2 |fold change| > 1 and FDR < 0.05). Then, the selected attributes function in the data mining tool WEKA [57] was used to obtain the final list of potential diagnostic biomarkers (nine Hypo-DMPs and one Hypo-DMP). Lastly, for machine learning in WEKA, we used BayesNet to construct the COAD-specific diagnostic model with the nine Hyper-DMPs and one Hypo-DMP in the training cohort from TCGA (including 200 COAD and 25 normal samples).

Then, the diagnostic model was verified with the validation cohort from TCGA (including 99 COAD and 13 normal samples) and five independent GEO COAD cohorts. Firstly, we imported the methylation values of the nine Hyper-DMPs and one Hypo-DMP from the training cohort using the *Filter* option of the WEKA *Preprocess* panel. Then, we selected BayesNet in the *Classifier* option of the *Classify* panel to build our model. Thereafter, we imported the methylation values of the nine Hyper-DMPs and one Hypo-DMP from the validation cohort (TCGA validation set or GSE42752 or GSE53051 or GSE77718 or GSE48684 or GSE77954) to the *Supplied test set* option of the *Classify* panel. Finally, selecting BayesNet in the *Classifier* option, we identified the sensitivity, specificity, and accuracy of our model in the evaluated cohort.

### Acquisition of candidate prognostic biomarkers and construction of a prognostic model

A total of 287 COAD tissue samples in the cohort from TCGA had both methylated *β* values and corresponding prognostic information. These samples were randomly assigned to the training cohort and the test cohort at a ratio of 1:1. Firstly, univariate Cox proportional hazard regression analysis was performed in the training cohort from TCGA (143 COAD samples) to identify significant methylation markers associated with the OS of COAD patients (*p* < 0.05). Then, multivariate Cox stepwise regression analysis was performed on these CpG sites, and sites with *p*-values > 0.05 were removed from the feature cohort in each iteration. The R packages *Survival* and *Mass* were jointly used to complete the multivariate Cox stepwise regression analysis.

The Cox proportional risk model was used to determine patients’ hazard ratios and corresponding 95% confidence intervals. The linear combination of model predictors weighted by their regression coefficients was used as the formula to predict the survival risk of patients. The high-risk and low-risk groups were classified according to the median risk value. The R packages *Survival* and *Plot* were then used to plot Kaplan-Meier survival curves to visualize the cumulative survival of the patients at risk at some time point. A log-rank test was used to evaluate the difference in OS between the high- and low-risk groups. Finally, the area under the ROC curve was determined by ROC analysis using the R package *SurvivalROC*. The effectiveness of the risk score in predicting OS was also evaluated. All statistical calculations were performed using the R statistical environment (R version 3.5.4).

### STRING database

The genes corresponding to the diagnostic model were analyzed using the STRING functional protein-protein interaction network (9.1) [58]. The same website was used to analyze the input GO biological processes and KEGG pathways. *P*-values < 0.05 were considered significant.

### GSEA

After the prognostic prediction model was used to calculate patients’ risk scores, GSEA (JAVA version) [59] (http://software.broadinstitute.org/gsea/index.jsp) was performed for the high-risk and low-risk groups. GSEA includes four key statistics: the ES, NES, FDR *q*-value and *p*-value. An ES > 0.4, |NES| > 1, *p*-value < 0.05 and FDR *q*-value < 0.25 were used to filter the GSEA results. Based on these statistics, all the genes in the list of specific genes were scored and ranked.

## Supplementary Material

Supplementary Figures

Supplementary Tables 1, 2, 3 and 4

Supplementary Table 5

Supplementary Table 6

Supplementary Tables 7 and 8
