# Supplementary material for: Colon cancer-specific diagnostic and prognostic biomarkers based on genome-wide abnormal DNA methylation
Source: Aging (Albany NY). 2020 Nov 17;12(22):22626–55. doi: 10.18632/aging.103874 (PMC7746390; doi:10.18632/aging.103874)
Supplement: Supplementary Figures [file aging-12-103874-s001..pdf]

SUPPLEMENTARY FIGURES

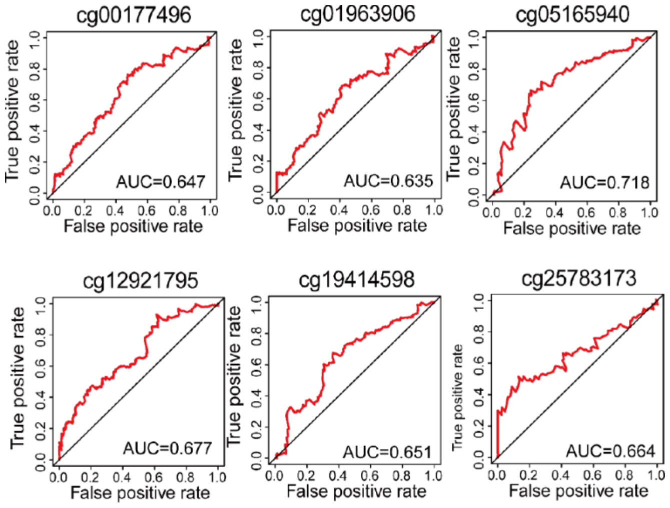

Supplementary Figure 1. The ROC curve analysis of 6 individual CpG site of prognostic biomarkers in the TCGA validation cohort (*N*=144).

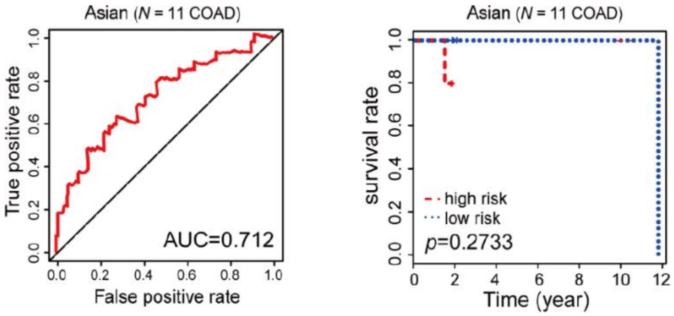

Supplementary Figure 2. Kaplan-Meier and ROC analysis results based on different regrouping methods. Grouping COAD patients according to race: Asian (N=11, 4.09%).

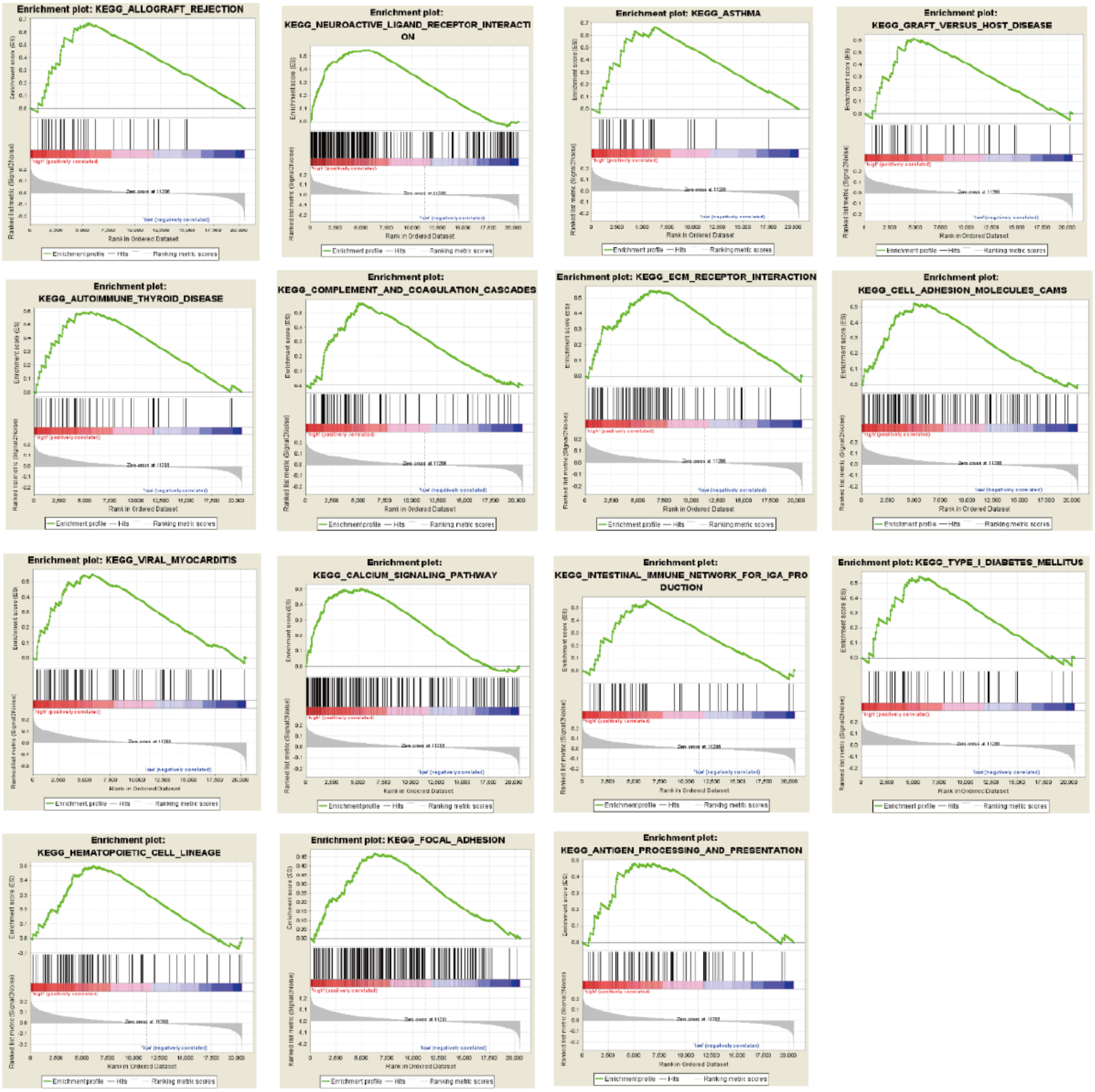

Supplementary Figure 3. The results of GSEA analysis with high-risk and low-risk groups from the TCGA COAD cohort (*N* = 287).

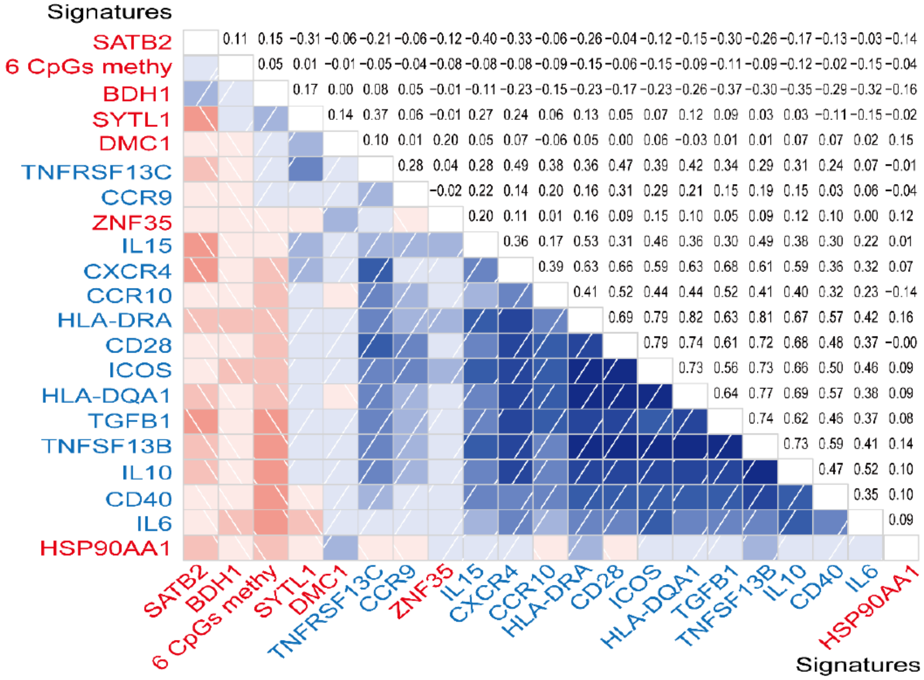

Supplementary Figure 4. Correlation between the genes corresponding to prognostic methylation biomarkers and the core enrichment genes of the intestinal immune network for the IGA production pathway. The circled area reflects Pearson's correlation coefficient. The red signature represents the expression value of the corresponding genes of 6 CpG sites and the 6-site combined methylation value; blue signature represents the expression of the core enrichment genes in the intestinal immune network for the IGA production pathway. Lower triangle: grids showing the correlation between two signatures; blue for positive correlation, red for the negative correlation. Upper triangle: numbers represent the one-to-one correlation coefficient, which increases uniformly as the correlation value moves away from 0; blue for positive correlation, red for the negative correlation. It is the digital form of the main diagram.
