# Supplementary material for: Colon cancer-specific diagnostic and prognostic biomarkers based on genome-wide abnormal DNA methylation
Source: Aging (Albany NY). 2020 Nov 17;12(22):22626–55. doi: 10.18632/aging.103874 (PMC7746390; doi:10.18632/aging.103874)
Supplement: Supplementary Tables 1, 2, 3 and 4 [file aging-12-103874-s002..pdf]

SUPPLEMENTARY TABLES

Supplementary Table 1. The result of correlation analysis between the methylation level of diagnostic biomarkers and the expression of corresponding genes (*N*=10).

Probe	Gene name	Cor-Value	FDR	Type
cg26036626	FBLIM1	-0.862	1.79e-15	Hyper-DMP
cg03882585	SYNE1	-0.579	1.29e-05	Hyper-DMP
cg08130988	EFEMP1	-0.539	6.49e-05	Hyper-DMP
cg16733654	PTPRS	-0.715	7.65e-09	Hyper-DMP
cg12587766	LIFR	-0.786	2.19e-11	Hyper-DMP
cg08808128	CLIP4	-0.723	4.48e-09	Hyper-DMP
cg13004587	SCGB3A1	-0.288	0.0448	Hyper-DMP
cg05038216	CLIP4	-0.735	1.82e-09	Hyper-DMP
cg09746736	SLC6A2	-0.699	2.38e-08	Hyper-DMP
cg26718707	DIP2C	0.362	0.0107	Hypo-DMP

Supplementary Table 2. The result of the KEGG pathway analysis about diagnostic biomarkers by STRING database.

Category	Term ID	Count	Term description	*p* value	Genes	Rich Factor
KEGG_PATHWAY	hsa05132	3	Salmonella infection	0.0007	FLNA, FLNB, FLNC	0.035714286
KEGG_PATHWAY	hsa04630	3	Jak-STAT signaling pathway	0.0022	CNTF, LIF, LIFR	0.01875
KEGG_PATHWAY	hsa04510	3	Focal adhesion	0.0026	FLNA, FLNB, FLNC	0.015228426
KEGG_PATHWAY	hsa05205	3	Proteoglycans in cancer	0.0026	FLNA, FLNB, FLNC	0.015384615
KEGG_PATHWAY	hsa04060	3	Cytokine-cytokine receptor interaction	0.0037	CNTF, LIF, LIFR	0.011406844
KEGG_PATHWAY	hsa04010	3	MAPK signaling pathway	0.0042	FLNA, FLNB, FLNC	0.010238908
KEGG_PATHWAY	hsa04550	2	Signaling pathways regulating pluripotency of stem cells	0.0103	LIF, LIFR	0.014492754

Supplementary Table 3. The result of gene ontology (GO) analysis about diagnostic biomarkers by STRING database.

Category	ID	Term	Genes	*p* value
BP	GO:0045185	maintenance of protein location	FLNA, FLNB, SUN1, SUN2, SYNE1	0.000013
BP	GO:0090292	nuclear matrix anchoring at nuclear membrane	SUN1, SUN2, SYNE1	0.000013
BP	GO:0090286	cytoskeletal anchoring at nuclear membrane	SUN1, SUN2, SYNE1	0.0000268
BP	GO:0032507	maintenance of protein location in cell	FLNB, SUN1, SUN2, SYNE1	0.0000695
BP	GO:0021987	cerebral cortex development	FLNA, PTPRS, SUN1, SUN2	0.00036
BP	GO:0021817	nucleokinesis involved in cell motility in cerebral cortex radial glia guided migration	SUN1, SUN2	0.00066
BP	GO:0034329	cell junction assembly	FBLIM1, FERMT2, FLNA, FLNC	0.00066
BP	GO:0048861	leukemia inhibitory factor signaling pathway	LIF, LIFR	0.0008
BP	GO:0022603	regulation of anatomical structure morphogenesis	CNTF, FBLIM1, FERMT2, FLNA, LIF, PTPRS, SCGB3A1	0.001
BP	GO:0070120	ciliary neurotrophic factor-mediated signaling pathway	CNTF, LIFR	0.001

Supplementary Table 4. The result of correlation analysis between the methylation level of prognostic biomarkers and the expression of corresponding genes (*N*=6).

Probe	Gene name	Cor-Value	FDR
cg00177496	BDH1	-0.246	2.541e-05
cg01963906	SYTL1	0.153	2.996e-02
cg05165940	SATB2	-0.448	1.76e-16
cg12921795	WDR20	-0.116	7.72e-04
cg19414598	DMC1	-0.465	9.708e-17
cg25783173	ZNF35	-0.258	4.496e-06
