# Supplementary material for: Colon cancer-specific diagnostic and prognostic biomarkers based on genome-wide abnormal DNA methylation
Source: Aging (Albany NY). 2020 Nov 17;12(22):22626–55. doi: 10.18632/aging.103874 (PMC7746390; doi:10.18632/aging.103874)
Supplement: Supplementary Table 5 [file aging-12-103874-s003..docx]

**Supplementary Table 5.** **The OS of patients in the low-risk versus high-risk groups for the training cohort of TCGA COAD dataset (*N* = 143).**

| **Id** | **Time(years)** | **States** | **Risk Score** | **Risk Type** |
| --- | --- | --- | --- | --- |
| TCGA-3L-AA1B-01 | 1.301369863 | 0 | 0.3748062 | low |
| TCGA-5M-AATE-01 | 3.287671233 | 0 | 0.2072394 | low |
| TCGA-A6-2671-01 | 3.646575342 | 1 | 1.3310232 | high |
| TCGA-A6-2681-01 | 3.8 | 0 | 0.3162384 | low |
| TCGA-A6-2682-01 | 1.161643836 | 1 | 0.3031783 | low |
| TCGA-A6-2684-01 | 3.087671233 | 0 | 0.840916 | high |
| TCGA-A6-5659-01 | 2.536986301 | 0 | 0.1878167 | low |
| TCGA-A6-5661-01 | 2.794520548 | 0 | 0.5431827 | high |
| TCGA-A6-5664-01 | 1.84109589 | 0 | 0.0641538 | low |
| TCGA-A6-5667-01 | 2.430136986 | 0 | 0.4512315 | high |
| TCGA-A6-6137-01 | 2.257534247 | 0 | 0.2676031 | low |
| TCGA-A6-6140-01 | 2.010958904 | 0 | 1.312198 | high |
| TCGA-A6-6141-01 | 0.356164384 | 0 | 0.0285398 | low |
| TCGA-A6-6142-01 | 2.090410959 | 0 | 0.1142852 | low |
| TCGA-A6-6649-01 | 2.01369863 | 0 | 0.413234 | low |
| TCGA-A6-6650-01 | 1.717808219 | 0 | 0.3281496 | low |
| TCGA-A6-6780-01 | 1.676712329 | 0 | 0.9904617 | high |
| TCGA-A6-6781-01 | 1.638356164 | 0 | 0.3941286 | low |
| TCGA-A6-A566-01 | 2.076712329 | 1 | 0.2356938 | low |
| TCGA-A6-A56B-01 | 4.687671233 | 1 | 0.3636359 | low |
| TCGA-A6-A5ZU-01 | 0.802739726 | 0 | 6.261968 | high |
| TCGA-AA-3489-01 | 0.58630137 | 1 | 5.3599084 | high |
| TCGA-AA-3492-01 | 0.252054795 | 1 | 190.88115 | high |
| TCGA-AA-3494-01 | 0.084931507 | 0 | 0.0404847 | low |
| TCGA-AA-3496-01 | 0.084931507 | 0 | 0.9281345 | high |
| TCGA-AA-3502-01 | 2.917808219 | 0 | 0.0476061 | low |
| TCGA-AA-3506-01 | 4.835616438 | 0 | 0.1180152 | low |
| TCGA-AA-3655-01 | 5.084931507 | 0 | 0.0891636 | low |
| TCGA-AA-3660-01 | 6.506849315 | 0 | 0.2204374 | low |
| TCGA-AA-3697-01 | 7.087671233 | 0 | 0.0947534 | low |
| TCGA-AA-3713-01 | 1.58630137 | 0 | 7.0253725 | high |
| TCGA-AD-6889-01 | 6.936986301 | 1 | 0.6726848 | high |
| TCGA-AD-6895-01 | 2.090410959 | 0 | 0.7259211 | high |
| TCGA-AD-6964-01 | 0.906849315 | 1 | 0.3115863 | low |
| TCGA-AY-5543-01 | 2.750684932 | 0 | 6.2253254 | high |
| TCGA-AY-A69D-01 | 1.487671233 | 0 | 0.1736867 | low |
| TCGA-AZ-4308-01 | 9.106849315 | 0 | 1.0947934 | high |
| TCGA-AZ-4313-01 | 6.328767123 | 0 | 0.1386154 | low |
| TCGA-AZ-4315-01 | 4.865753425 | 0 | 0.0329266 | low |
| TCGA-AZ-4614-01 | 0.471232877 | 1 | 12.386297 | high |
| TCGA-AZ-4615-01 | 2.745205479 | 0 | 0.2841241 | low |
| TCGA-AZ-4682-01 | 1.863013699 | 1 | 0.9292691 | high |
| TCGA-AZ-6598-01 | 4.117808219 | 1 | 4.9432744 | high |
| TCGA-AZ-6599-01 | 0.564383562 | 1 | 0.1251059 | low |
| TCGA-AZ-6601-01 | 8.334246575 | 1 | 0.2924903 | low |
| TCGA-AZ-6605-01 | 0.435616438 | 1 | 0.7936851 | high |
| TCGA-AZ-6606-01 | 0.978082192 | 1 | 0.451122 | high |
| TCGA-CA-5256-01 | 1.038356164 | 0 | 3.7757466 | high |
| TCGA-CA-5796-01 | 1.032876712 | 0 | 0.6748804 | high |
| TCGA-CA-6719-01 | 1.191780822 | 0 | 2.333922 | high |
| TCGA-CK-4947-01 | 1.463013699 | 0 | 7.9615354 | high |
| TCGA-CK-4950-01 | 7.120547945 | 0 | 0.3536142 | low |
| TCGA-CK-4952-01 | 1.301369863 | 0 | 0.3646323 | low |
| TCGA-CK-5914-01 | 0.832876712 | 0 | 0.0320931 | low |
| TCGA-CK-6747-01 | 6.912328767 | 0 | 0.0764773 | low |
| TCGA-CK-6748-01 | 0.15890411 | 0 | 0.1724208 | low |
| TCGA-CK-6751-01 | 10.35616438 | 0 | 0.0588446 | low |
| TCGA-CM-4747-01 | 2.084931507 | 0 | 0.1586042 | low |
| TCGA-CM-4748-01 | 2.169863014 | 0 | 0.1035245 | low |
| TCGA-CM-4750-01 | 0.668493151 | 0 | 0.0908031 | low |
| TCGA-CM-5341-01 | 2.421917808 | 0 | 0.4707662 | high |
| TCGA-CM-5344-01 | 1.835616438 | 0 | 1.1942365 | high |
| TCGA-CM-5348-01 | 1.915068493 | 0 | 3.216846 | high |
| TCGA-CM-5349-01 | 2.506849315 | 0 | 0.8486009 | high |
| TCGA-CM-5861-01 | 1.252054795 | 0 | 3.2008197 | high |
| TCGA-CM-5862-01 | 0.419178082 | 1 | 26.245632 | high |
| TCGA-CM-5863-01 | 1.252054795 | 0 | 0.7953007 | high |
| TCGA-CM-5864-01 | 1.252054795 | 0 | 1.9093214 | high |
| TCGA-CM-6162-01 | 1 | 0 | 0.2172533 | low |
| TCGA-CM-6164-01 | 2.419178082 | 0 | 0.1918952 | low |
| TCGA-CM-6165-01 | 1.336986301 | 0 | 0.6747151 | high |
| TCGA-CM-6166-01 | 1.832876712 | 0 | 0.2853347 | low |
| TCGA-CM-6171-01 | 1.169863014 | 0 | 1.8578691 | high |
| TCGA-CM-6172-01 | 0.917808219 | 0 | 0.7502085 | high |
| TCGA-CM-6675-01 | 1.087671233 | 0 | 3.6745985 | high |
| TCGA-CM-6677-01 | 0.923287671 | 0 | 0.3564686 | low |
| TCGA-CM-6678-01 | 0.917808219 | 0 | 0.0809689 | low |
| TCGA-CM-6680-01 | 1.002739726 | 0 | 0.044203 | low |
| TCGA-D5-5538-01 | 4.550684932 | 1 | 0.4428174 | high |
| TCGA-D5-5539-01 | 1.632876712 | 0 | 0.0555382 | low |
| TCGA-D5-5540-01 | 4.673972603 | 0 | 0.723087 | high |
| TCGA-D5-6529-01 | 1.682191781 | 0 | 0.1640969 | low |
| TCGA-D5-6533-01 | 2.123287671 | 0 | 0.259606 | low |
| TCGA-D5-6534-01 | 3.605479452 | 0 | 0.6241966 | high |
| TCGA-D5-6536-01 | 1.487671233 | 0 | 0.0334591 | low |
| TCGA-D5-6537-01 | 0.4 | 1 | 0.3812459 | low |
| TCGA-D5-6538-01 | 1.42739726 | 0 | 0.6076815 | high |
| TCGA-D5-6539-01 | 1.04109589 | 0 | 0.0734636 | low |
| TCGA-D5-6540-01 | 1.345205479 | 0 | 0.4764264 | high |
| TCGA-D5-6541-01 | 1.298630137 | 0 | 0.2865816 | low |
| TCGA-D5-6898-01 | 0.62739726 | 0 | 0.2742456 | low |
| TCGA-D5-6922-01 | 0.843835616 | 0 | 0.1719604 | low |
| TCGA-D5-6924-01 | 1.191780822 | 0 | 0.5692247 | high |
| TCGA-D5-6928-01 | 0.969863014 | 0 | 0.6595033 | high |
| TCGA-D5-6929-01 | 1.117808219 | 0 | 0.6318862 | high |
| TCGA-D5-6930-01 | 1.112328767 | 0 | 0.4302965 | low |
| TCGA-D5-6931-01 | 1 | 0 | 0.1016847 | low |
| TCGA-D5-6932-01 | 0.947945205 | 0 | 0.1509736 | low |
| TCGA-DM-A0XD-01 | 2.035616438 | 1 | 1.4478311 | high |
| TCGA-DM-A1D0-01 | 10.88767123 | 0 | 0.022357 | low |
| TCGA-DM-A1D4-01 | 7.728767123 | 1 | 0.5238085 | high |
| TCGA-DM-A1D7-01 | 1.109589041 | 1 | 0.4660767 | high |
| TCGA-DM-A1D9-01 | 11.69863014 | 0 | 0.0628682 | low |
| TCGA-DM-A1DA-01 | 0.624657534 | 1 | 9.0642577 | high |
| TCGA-DM-A1HB-01 | 11.30410959 | 0 | 0.0708225 | low |
| TCGA-DM-A280-01 | 0.646575342 | 1 | 1.5957164 | high |
| TCGA-DM-A285-01 | 0.490410959 | 1 | 3.9904082 | high |
| TCGA-DM-A288-01 | 1.169863014 | 1 | 0.9960656 | high |
| TCGA-DM-A28A-01 | 2.205479452 | 1 | 1.174093 | high |
| TCGA-DM-A28E-01 | 9.994520548 | 0 | 0.6419805 | high |
| TCGA-DM-A28F-01 | 2.997260274 | 1 | 1.9676357 | high |
| TCGA-DM-A28G-01 | 5.065753425 | 1 | 0.2734171 | low |
| TCGA-DM-A28H-01 | 9.756164384 | 0 | 0.4028619 | low |
| TCGA-F4-6460-01 | 2.663013699 | 1 | 4.1434315 | high |
| TCGA-F4-6570-01 | 0.515068493 | 1 | 17.661224 | high |
| TCGA-F4-6704-01 | 0.128767123 | 0 | 0.1773854 | low |
| TCGA-F4-6806-01 | 3.452054795 | 0 | 0.8964568 | high |
| TCGA-F4-6807-01 | 3.58630137 | 0 | 0.1977086 | low |
| TCGA-F4-6809-01 | 1.104109589 | 1 | 0.2893515 | low |
| TCGA-F4-6856-01 | 2.942465753 | 0 | 0.2291523 | low |
| TCGA-G4-6297-01 | 6.865753425 | 0 | 0.2779427 | low |
| TCGA-G4-6298-01 | 1.95890411 | 1 | 1.7785031 | high |
| TCGA-G4-6299-01 | 6.21369863 | 0 | 0.2126581 | low |
| TCGA-G4-6302-01 | 5.608219178 | 1 | 2.5059227 | high |
| TCGA-G4-6303-01 | 5.487671233 | 1 | 0.9893366 | high |
| TCGA-G4-6304-01 | 4.468493151 | 0 | 0.023712 | low |
| TCGA-G4-6309-01 | 7.123287671 | 0 | 0.0758288 | low |
| TCGA-G4-6310-01 | 5.301369863 | 0 | 1.1056469 | high |
| TCGA-G4-6311-01 | 3.284931507 | 0 | 0.0042397 | low |
| TCGA-G4-6315-01 | 5.15890411 | 0 | 0.4279042 | low |
| TCGA-G4-6317-01 | 3 | 0 | 0.5804231 | high |
| TCGA-G4-6317-02 | 3 | 0 | 0.6289722 | high |
| TCGA-G4-6321-01 | 1.84109589 | 0 | 0.0434376 | low |
| TCGA-G4-6586-01 | 2.983561644 | 0 | 0.2036231 | low |
| TCGA-G4-6588-01 | 2.180821918 | 0 | 0.4344127 | high |
| TCGA-G4-6625-01 | 7.649315068 | 0 | 0.0414683 | low |
| TCGA-G4-6627-01 | 6.232876712 | 0 | 0.7526513 | high |
| TCGA-G4-6628-01 | 6.64109589 | 0 | 1.0183393 | high |
| TCGA-NH-A50T-01 | 1.515068493 | 0 | 2.6351342 | high |
| TCGA-NH-A50V-01 | 1.610958904 | 0 | 0.9824318 | high |
| TCGA-NH-A6GA-01 | 0.82739726 | 1 | 9.4327287 | high |
| TCGA-NH-A6GB-01 | 1.304109589 | 0 | 2.0143828 | high |
| TCGA-NH-A8F8-01 | 1.4 | 1 | 1.8830328 | high |
