# Supplementary material for: Colon cancer-specific diagnostic and prognostic biomarkers based on genome-wide abnormal DNA methylation
Source: Aging (Albany NY). 2020 Nov 17;12(22):22626–55. doi: 10.18632/aging.103874 (PMC7746390; doi:10.18632/aging.103874)
Supplement: Supplementary Table 6 [file aging-12-103874-s004..docx]

**Supplementary Table 6. The OS of patients in the low-risk versus high-risk groups for the validation cohort of TCGA COAD dataset (*N* = 144).**

| **Id** | **Time(years)** | **States** | **Risk Score** | **Risk Type** |
| --- | --- | --- | --- | --- |
| TCGA-4N-A93T-01 | 0.4 | 0 | 0.4833259 | high |
| TCGA-4T-AA8H-01 | 1.054795 | 0 | 4.4911912 | high |
| TCGA-5M-AAT4-01 | 0.134247 | 1 | 0.8769674 | high |
| TCGA-5M-AAT6-01 | 0.794521 | 1 | 0.7026028 | high |
| TCGA-A6-2672-01 | 3.887671 | 0 | 2.6235612 | high |
| TCGA-A6-2675-01 | 3.619178 | 0 | 0.0336437 | low |
| TCGA-A6-2677-01 | 2.027397 | 1 | 0.3702038 | low |
| TCGA-A6-2680-01 | 2.926027 | 0 | 0.2269892 | low |
| TCGA-A6-2685-01 | 3.10411 | 0 | 2.1210337 | high |
| TCGA-A6-2686-01 | 3.084932 | 1 | 12.099006 | high |
| TCGA-A6-3809-01 | 2.728767 | 0 | 1.2275955 | high |
| TCGA-A6-3810-01 | 3.043836 | 0 | 0.6044333 | high |
| TCGA-A6-4105-01 | 1.210959 | 1 | 0.0546698 | low |
| TCGA-A6-4107-01 | 2.70411 | 0 | 4.3584662 | high |
| TCGA-A6-5656-01 | 2.742466 | 0 | 0.3293683 | low |
| TCGA-A6-5657-01 | 2.635616 | 0 | 0.9412076 | high |
| TCGA-A6-5660-01 | 2.432877 | 0 | 0.1095112 | low |
| TCGA-A6-5662-01 | 1.967123 | 0 | 0.113057 | low |
| TCGA-A6-5665-01 | 1.838356 | 0 | 0.4388644 | high |
| TCGA-A6-5666-01 | 2.726027 | 0 | 0.5755401 | high |
| TCGA-A6-6138-01 | 1.876712 | 0 | 0.0613796 | low |
| TCGA-A6-6648-01 | 2.09863 | 0 | 0.6828014 | high |
| TCGA-A6-6651-01 | 1.813699 | 0 | 1.7504385 | high |
| TCGA-A6-6652-01 | 2.057534 | 0 | 0.1875325 | low |
| TCGA-A6-6653-01 | 2.032877 | 0 | 0.3452441 | low |
| TCGA-A6-6654-01 | 1.989041 | 0 | 6.9646449 | high |
| TCGA-A6-6782-01 | 1.690411 | 0 | 1.2427336 | high |
| TCGA-A6-A565-01 | 1.353425 | 1 | 2.5431453 | high |
| TCGA-A6-A567-01 | 5.153425 | 1 | 0.9278566 | high |
| TCGA-AA-3488-01 | 0.419178 | 1 | 5.0712216 | high |
| TCGA-AA-3495-01 | 3.087671 | 0 | 0.1846152 | low |
| TCGA-AA-3509-01 | 5.246575 | 0 | 0.0224409 | low |
| TCGA-AA-3510-01 | 5.331507 | 0 | 0.0466319 | low |
| TCGA-AA-3511-01 | 0.580822 | 0 | 0.362203 | low |
| TCGA-AA-3662-01 | 0.50411 | 0 | 0.0468608 | low |
| TCGA-AA-3663-01 | 0.580822 | 0 | 5.2937117 | high |
| TCGA-AD-5900-01 | 1.013699 | 0 | 2.9646319 | high |
| TCGA-AD-6548-01 | 1.780822 | 0 | 0.504681 | high |
| TCGA-AD-6888-01 | 1.293151 | 1 | 1.0177063 | high |
| TCGA-AD-6890-01 | 2.043836 | 0 | 1.1963096 | high |
| TCGA-AD-6899-01 | 0.482192 | 1 | 1.5367626 | high |
| TCGA-AD-6901-01 | 1.868493 | 1 | 1.5003705 | high |
| TCGA-AD-6963-01 | 2.284932 | 0 | 0.2979301 | low |
| TCGA-AD-6965-01 | 2.205479 | 0 | 0.0650819 | low |
| TCGA-AD-A5EK-01 | 1.369863 | 0 | 0.3831282 | low |
| TCGA-AM-5820-01 | 0.038356 | 0 | 0.0690772 | low |
| TCGA-AM-5821-01 | 0.076712 | 0 | 7.0156567 | high |
| TCGA-AU-6004-01 | 2.257534 | 0 | 2.9722998 | high |
| TCGA-AY-6197-01 | 1.786301 | 0 | 1.4864562 | high |
| TCGA-AY-6386-01 | 1.484932 | 0 | 0.0277226 | low |
| TCGA-AY-A54L-01 | 1.438356 | 0 | 0.2387265 | low |
| TCGA-AY-A71X-01 | 1.610959 | 0 | 0.4297558 | high |
| TCGA-AY-A8YK-01 | 1.569863 | 0 | 11.164333 | high |
| TCGA-AZ-4323-01 | 0.117808 | 1 | 0.4552239 | high |
| TCGA-AZ-4616-01 | 0.427397 | 1 | 0.1231453 | low |
| TCGA-AZ-4681-01 | 8.89589 | 0 | 0.194388 | low |
| TCGA-AZ-4684-01 | 5.416438 | 0 | 0.0743355 | low |
| TCGA-AZ-5403-01 | 5.232877 | 1 | 0.7353415 | high |
| TCGA-AZ-5407-01 | 7.350685 | 0 | 0.260789 | low |
| TCGA-AZ-6603-01 | 2.463014 | 1 | 65.205335 | high |
| TCGA-AZ-6607-01 | 0.265753 | 1 | 6642.7586 | high |
| TCGA-AZ-6608-01 | 0.161644 | 1 | 1.0166286 | high |
| TCGA-CA-5254-01 | 1.057534 | 0 | 0.1221905 | low |
| TCGA-CA-5255-01 | 1.030137 | 0 | 0.4784136 | high |
| TCGA-CA-5797-01 | 1.049315 | 0 | 0.7633117 | high |
| TCGA-CA-6715-01 | 1.049315 | 0 | 0.275615 | low |
| TCGA-CA-6716-01 | 1.016438 | 0 | 0.2944096 | low |
| TCGA-CA-6717-01 | 1.063014 | 0 | 0.1632318 | low |
| TCGA-CA-6718-01 | 0.838356 | 1 | 516.9342 | high |
| TCGA-CK-4948-01 | 12.33425 | 0 | 0.0819705 | low |
| TCGA-CK-4951-01 | 5.846575 | 1 | 0.0600924 | low |
| TCGA-CK-5912-01 | 4.090411 | 1 | 76.47783 | high |
| TCGA-CK-5913-01 | 4.276712 | 0 | 1.1400079 | high |
| TCGA-CK-5916-01 | 1.761644 | 1 | 5.6207801 | high |
| TCGA-CM-4743-01 | 1.920548 | 0 | 0.2468092 | low |
| TCGA-CM-4744-01 | 1.668493 | 0 | 0.1660157 | low |
| TCGA-CM-4746-01 | 3.084932 | 0 | 0.0154435 | low |
| TCGA-CM-4751-01 | 2.252055 | 0 | 1.9648136 | high |
| TCGA-CM-4752-01 | 1.084932 | 0 | 0.1575794 | low |
| TCGA-CM-5860-01 | 2.668493 | 0 | 0.3092996 | low |
| TCGA-CM-5868-01 | 1.419178 | 0 | 1.738855 | high |
| TCGA-CM-6161-01 | 1.252055 | 0 | 1.345217 | high |
| TCGA-CM-6163-01 | 1.169863 | 0 | 1.2290754 | high |
| TCGA-CM-6167-01 | 1.249315 | 0 | 0.5048633 | high |
| TCGA-CM-6168-01 | 1.082192 | 0 | 1.6085515 | high |
| TCGA-CM-6169-01 | 1.084932 | 0 | 0.0123564 | low |
| TCGA-CM-6170-01 | 1.252055 | 0 | 0.0829949 | low |
| TCGA-CM-6674-01 | 1.079452 | 0 | 0.3645044 | low |
| TCGA-CM-6676-01 | 0.923288 | 0 | 0.967682 | high |
| TCGA-CM-6679-01 | 0.838356 | 0 | 0.1612318 | low |
| TCGA-D5-5537-01 | 3.783562 | 1 | 0.6856107 | high |
| TCGA-D5-5541-01 | 4.660274 | 0 | 0.1706113 | low |
| TCGA-D5-6530-01 | 1.70137 | 0 | 0.2410849 | low |
| TCGA-D5-6531-01 | 1.479452 | 0 | 0.2837999 | low |
| TCGA-D5-6532-01 | 1.520548 | 0 | 0.1018079 | low |
| TCGA-D5-6535-01 | 1.260274 | 0 | 0.1811235 | low |
| TCGA-D5-6920-01 | 1.032877 | 0 | 0.0820607 | low |
| TCGA-D5-6923-01 | 1.035616 | 0 | 2.4041205 | high |
| TCGA-D5-6926-01 | 0.753425 | 0 | 0.3164449 | low |
| TCGA-D5-7000-01 | 0.854795 | 0 | 0.2216878 | low |
| TCGA-DM-A0X9-01 | 9.975342 | 0 | 0.1224407 | low |
| TCGA-DM-A0XF-01 | 3.183562 | 1 | 0.8447837 | high |
| TCGA-DM-A1D6-01 | 4.158904 | 1 | 1.2750148 | high |
| TCGA-DM-A1D8-01 | 1.049315 | 1 | 0.2667232 | low |
| TCGA-DM-A1DB-01 | 3.693151 | 1 | 0.4610682 | high |
| TCGA-DM-A1HA-01 | 10.9589 | 0 | 0.0370354 | low |
| TCGA-DM-A282-01 | 11.59726 | 0 | 0.1008705 | low |
| TCGA-DM-A28C-01 | 6.780822 | 1 | 0.1230536 | low |
| TCGA-DM-A28K-01 | 8.186301 | 0 | 0.1912917 | low |
| TCGA-DM-A28M-01 | 7.931507 | 0 | 0.077092 | low |
| TCGA-F4-6459-01 | 0.717808 | 1 | 0.2004692 | low |
| TCGA-F4-6461-01 | 0.926027 | 1 | 14.584084 | high |
| TCGA-F4-6463-01 | 2.978082 | 0 | 0.5172373 | high |
| TCGA-F4-6569-01 | 2.978082 | 0 | 6.4140781 | high |
| TCGA-F4-6703-01 | 3.989041 | 0 | 0.4181059 | high |
| TCGA-F4-6805-01 | 2.868493 | 0 | 0.2589291 | low |
| TCGA-F4-6808-01 | 2.805479 | 0 | 0.3105939 | low |
| TCGA-F4-6854-01 | 0.043836 | 0 | 0.7594359 | high |
| TCGA-F4-6855-01 | 3.950685 | 0 | 0.4621109 | high |
| TCGA-G4-6293-01 | 11.09863 | 0 | 0.028908 | low |
| TCGA-G4-6294-01 | 2.350685 | 1 | 0.4965754 | high |
| TCGA-G4-6295-01 | 0.69589 | 0 | 0.3792163 | low |
| TCGA-G4-6306-01 | 3.723288 | 0 | 0.677758 | high |
| TCGA-G4-6307-01 | 4.586301 | 0 | 0.0186541 | low |
| TCGA-G4-6314-01 | 2.994521 | 0 | 1.3858619 | high |
| TCGA-G4-6320-01 | 2.20274 | 0 | 0.1302272 | low |
| TCGA-G4-6322-01 | 2.169863 | 0 | 0.6991681 | high |
| TCGA-G4-6323-01 | 1.147945 | 0 | 0.1543227 | low |
| TCGA-G4-6626-01 | 3.89589 | 1 | 0.5233981 | high |
| TCGA-NH-A50U-01 | 0.915068 | 1 | 0.0378292 | low |
| TCGA-NH-A5IV-01 | 1.610959 | 0 | 0.0560274 | low |
| TCGA-NH-A6GC-01 | 1.065753 | 0 | 0.4606093 | high |
| TCGA-NH-A8F7-01 | 1.487671 | 0 | 2.7204359 | high |
| TCGA-NH-A8F7-06 | 1.487671 | 0 | 0.3642142 | low |
| TCGA-QG-A5YV-01 | 3.564384 | 0 | 0.0681469 | low |
| TCGA-QG-A5YW-01 | 2.454795 | 0 | 0.0356898 | low |
| TCGA-QG-A5YX-01 | 2.747945 | 0 | 0.2178051 | low |
| TCGA-QG-A5Z1-01 | 0.70137 | 1 | 73.540646 | high |
| TCGA-QG-A5Z2-01 | 2.608219 | 0 | 0.5660852 | high |
| TCGA-QL-A97D-01 | 1.824658 | 0 | 0.4272473 | high |
| TCGA-RU-A8FL-01 | 3.224658 | 0 | 0.2839154 | low |
| TCGA-SS-A7HO-01 | 5.010959 | 0 | 0.4038222 | low |
| TCGA-T9-A92H-01 | 0.991781 | 0 | 0.0880929 | low |
| TCGA-WS-AB45-01 | 5.835616 | 0 | 11.429489 | high |
