# Supplementary material for: Colon cancer-specific diagnostic and prognostic biomarkers based on genome-wide abnormal DNA methylation
Source: Aging (Albany NY). 2020 Nov 17;12(22):22626–55. doi: 10.18632/aging.103874 (PMC7746390; doi:10.18632/aging.103874)
Supplement: Supplementary Tables 7 and 8 [file aging-12-103874-s005..pdf]

Supplementary Table 7. The result of the KEGG pathway analysis about prognostic biomarkers by GSEA analysis.

GS<br>follow link to MSigDB	SIZE	ES	NES	NOM *p*-val	FDR *q*-val	FWER *p*-val	RANK AT MAX	LEADING EDGE
KEGG_ALLOGRAFT_REJECTION	35	0.67	1.78	0	0.0083	0.011	5550	tags=66%, list=27%, signal=90%
KEGG_NEUROACTIVE_LIGAND_RECEPTOR_INTERACTION	271	0.54	1.71	0	0.0200	0.053	5386	tags=61%, list=26%, signal=81%
KEGG_ASTHMA	28	0.66	1.70	0.0048	0.0196	0.078	6188	tags=82%, list=30%, signal=117%
KEGG_GRAFT_VERSUS_HOST_DISEASE	37	0.62	1.66	0	0.0287	0.145	4787	tags=59%, list=23%, signal=77%
KEGG_AUTOIMMUNE_THYROID_DISEASE	50	0.60	1.65	0	0.0264	0.164	5550	tags=54%, list=27%, signal=74%
KEGG_COMPLEMENT_AND_COAGULATION_CASCADES	68	0.56	1.61	0	0.0420	0.284	5143	tags=59%, list=25%, signal=78%
KEGG_ECM_RECEPTOR_INTERACTION	83	0.54	1.59	0	0.0452	0.338	6223	tags=60%, list=30%, signal=86%
KEGG_CELL_ADHESION_MOLECULES_CAMS	131	0.52	1.58	0	0.0462	0.384	5014	tags=45%, list=24%, signal=59%
KEGG_VIRAL_MYOCARDITIS	68	0.55	1.56	0.0055	0.0518	0.455	5673	tags=51%, list=28%, signal=71%
KEGG_CALCIUM_SIGNALING_PATHWAY	177	0.50	1.54	0	0.0639	0.577	5290	tags=48%, list=26%, signal=64%
KEGG_INTESTINAL_IMMUNE_NETWORK_FOR_IGA_PRODUCTION	46	0.56	1.52	0.0090	0.0779	0.679	6188	tags=70%, list=30%, signal=99%
KEGG_TYPE_I_DIABETES_MELLITUS	41	0.55	1.47	0.0228	0.1358	0.897	5550	tags=56%, list=27%, signal=77%
KEGG_HEMATOPOIETIC_CELL_LINEAGE	84	0.50	1.45	0.0053	0.1564	0.944	6019	tags=62%, list=29%, signal=87%
KEGG_FOCAL_ADHESION	198	0.47	1.44	0.0010	0.1744	0.968	6302	tags=43%, list=31%, signal=62%
KEGG_ANTIGEN_PROCESSING_AND_PRESENTATION	81	0.48	1.40	0.0322	0.2397	0.993	4958	tags=33%, list=24%, signal=44%

Supplementary Table 8. The core enrichment genes of the intestinal immune network for IGA production by GSEA analysis.

NAME	PROBE	RANK IN GENE LIST	RANK METRIC SCORE	RUNNING ES	CORE ENRICHMENT
row_0	HLA-DRB5	758	0.123399086	0.018374009	Yes
row_1	HLA-DQB1	1158	0.109066784	0.047841907	Yes
row_2	IL6	1225	0.106780887	0.09254054	Yes
row_3	HLA-DRB1	1493	0.099003181	0.12393619	Yes
row_4	CCR9	1650	0.094628379	0.1587874	Yes
row_5	HLA-DOB	1681	0.093834162	0.19943333	Yes
row_6	CD40LG	1723	0.092663467	0.23901686	Yes
row_7	CD40	2092	0.084532633	0.2589878	Yes
row_8	HLA-DMA	2802	0.070332468	0.25593892	Yes
row_9	HLA-DQA1	2849	0.06942334	0.28484878	Yes
row_10	IL10	2862	0.069310784	0.31536794	Yes
row_11	IL15	3011	0.06663157	0.33804542	Yes
row_12	TNFSF13B	3177	0.06373629	0.35859364	Yes
row_13	HLA-DMB	3213	0.063404351	0.38533932	Yes
row_14	CXCL12	3547	0.05772195	0.39498693	Yes
row_15	CCR10	3651	0.05583854	0.41501758	Yes
row_16	CXCR4	3708	0.054980859	0.4369578	Yes
row_17	HLA-DRA	4037	0.049792189	0.44329083	Yes

row_18	HLA-DPA1	4090	0.049007826	0.4627458	Yes
row_19	HLA-DPB1	4236	0.046960581	0.47674185	Yes
row_20	MADCAM1	4634	0.041817173	0.47612742	Yes
row_21	CD28	4787	0.039844017	0.486588	Yes
row_22	TNFRSF13B	4805	0.039523255	0.50349516	Yes
row_23	TNFRSF13C	4824	0.039233908	0.5202236	Yes
row_24	IL5	5096	0.035951488	0.523128	Yes
row_25	CD86	5550	0.031060619	0.51495236	Yes
row_26	ICOS	5788	0.028889718	0.51634735	Yes
row_27	IL15RA	5898	0.02780547	0.52350456	Yes
row_28	ITGA4	6019	0.026598617	0.52958316	Yes
row_29	ITGB7	6124	0.025798159	0.5360836	Yes
row_30	TGFB1	6148	0.025601827	0.54645026	Yes
row_31	HLA-DOA	6188	0.025266264	0.5558852	Yes
